# Single-molecule magnetic tweezers to probe the equilibrium dynamics of individual proteins at physiologically-relevant forces and timescales

**DOI:** 10.1038/s41596-024-00965-5

**Published:** 2024-03-11

**Authors:** Rafael Tapia-Rojo, Marc Mora, Sergi Garcia-Manyes

**Affiliations:** 1 Single Molecule Mechanobiology Laboratory, The Francis Crick Institute, 1 Midland Road, London NW1 1AT, London, UK; 2 Department of Physics, Randall Centre for Cell and Molecular Biophysics, Centre for the Physical Science of Life and London Centre for Nanotechnology, King’s College London, Strand, WC2R 2LS London, United Kingdom

## Abstract

The reversible unfolding and refolding of proteins is a regulatory mechanism of tissue elasticity and signalling, used by cells to sense and adapt to extracellular and intracellular mechanical forces. However, most of these proteins exhibit low mechanical stability, posing technical challenges to the characterization of their conformational dynamics under force. Here, we detail the step-by-step instructions for conducting single protein nanomechanical experiments using ultra-stable magnetic tweezers, which enable the measurement of the equilibrium conformational dynamics of single proteins under physiologically-relevant low forces applied over biologically-relevant timescales. We report the basic principles determining the functioning of the magnetic tweezer instrument, review the protein design strategy and the fluid chamber preparation and detail the procedure to acquire and analyze the unfolding and refolding trajectories of individual proteins under force. This technique adds to the toolbox of single-molecule nanomechanical techniques and will be of particular interest to those interested in proteins involved in mechanosensing and mechanotransduction. The procedure takes 4 days to complete, plus 6 additional days for protein cloning and production, requiring basic expertise in molecular biology, surface chemistry and data analysis.

## Introduction

Mounting evidence demonstrates the crucial role that mechanical forces play in determining biological function. For example, mechanical forces regulate differentiation and cell fate^1,2^, shape^3^, motility^4^, proliferation, and growth^5,6^. They also underpin muscle contractility^7^, touch, blood-pressure sensing, and hearing^8^. More recently, mechanical forces have been directly linked to both the functioning of the immune system^9^ and to metabolism regulation^10^. The dysregulation of mechanical cues involved in homeostasis can further lead to cancer^11^ and to a variety of myopathies^12^. Therefore, a complete understanding of these mechanosensing and mechanotransduction processes requires unveiling the molecular mechanisms underpinning such cellular and tissue manifestations. In addition to well-studied force-dependent molecular systems such as ATP-dependent molecular motors^13^ and pressure-induced ion channels^8,14^, measuring the conformational responses of individual proteins under force provides a fundamental means to probe cell and tissue mechanobiology.

The two most common and complementary single-molecule manipulation techniques typically used to measure the force-induced conformational changes of proteins^15^ are the atomic force microscope (AFM)^16^ and optical tweezers^17^. Briefly, the AFM excels at determining the nanomechanical properties of mechanically stable proteins – that is, proteins requiring forces >60-100 pN to unfold^18^ such as Ig or fibronectin-like folds^19^ or cell surface adhesins^20,21^ – and at measuring their force-induced chemical reactivity^22^. In out-of-equilibrium experiments, the low-force behavior of proteins cannot be directly measured, and it is instead extrapolated from the experimental data conducted at higher forces (or pulling speeds) using an adequate unfolding rate-pulling force relationship^23^. At the other end of the force spectrum, optical tweezers have been employed to measure the unfolding and refolding dynamics of proteins when stretched in physiological, low force regimes (<20 pN)^17^. These experiments have enabled reconstruction of the folding energy landscape of relatively uncomplicated proteins^24–27^, and the forces involved during co-translational folding^28^. However, due to their inherent experimental mechanical drift, these measurements have been typically limited to probe minute-long timescales.

The challenge lies in directly measuring the equilibrium conformational dynamics of proteins at physiologically operating low forces and over physiologically-long timescales, encompassing many hours or even days, as a natural approach to begin to correlate the *in vitro* behavior of these proteins when exposed *in-vivo* to physiological mechanical forces inside the cell. Toward this goal, single-molecule magnetic tweezers, traditionally applied to measure the mechanical properties of individual long nucleic acid molecules^29–32^, have recently begun to be applied to the measurement of protein nanomechanics, including soluble^33–39^ and membrane proteins^40–42^. Thanks to their intrinsic temporal stability and ability to accurately manipulate low pulling forces—combined with recent developments in instrumentation and protein tethering strategies—, single-molecule magnetic tweezers is emerging as a promising technique for those aiming to study protein mechanobiology.

Herein, we comprehensively report on the different steps required to prepare a single protein experiment using magnetic tweezers force spectroscopy, focusing on the force calibration, sample preparation, single-molecule measurements, and subsequent data analysis.

## Development and application of the protocol

Magnetic tweezers were developed in the 1990s and its use extended during the ‘2000s, mostly to study the elastic properties of long DNA tethers^43–48^. While the initial notion of single-molecule magnetic tweezers instrumentation has remained fundamentally unchanged, a combination of *(i)* significant instrument development, *(ii)* the design of covalent-anchoring techniques^49,50^, and *(iii)* a suitable calibration method based on the elasticity of short proteins^37^ has recently enabled the extension of its applicability to the measurement of protein nanomechanics^34,37,38,40,51^. These technological improvements have enabled us to obtain ultra-stable recordings, lasting several days and even weeks, on the same protein as it unfolds and refolds under force in equilibrium^52–54^. Significantly increasing the experimental measuring window has enabled us to gain access to remote regions of the protein’s folding free energy landscape, discovering low-probability conformations that, besides being structurally fingerprinted through fluctuation analysis, might hold biological function *in-vivo*^55^.

These experiments are opening the door to routinely studying the equilibrium unfolding and refolding dynamics of a wide variety of mechanosensing proteins that naturally operate at very low forces^56^. Within the cellular context, mechanical tensions of only a few piconewtons (pN), as revealed by fluorescence force sensors^56–58^, trigger conformational changes that lead those force-bearing proteins to unfold. Probing their molecular dynamics under force *in vitro* will now enable a mechanistic understanding of the strategies employed by each mechanosensor to reversibly propagate, buffer, absorb, store or release energy through mechanical unfolding and refolding, providing a molecular vista on the mechanisms at play during cellular mechanostransduction. As such, many proteins at the focal adhesion, cell-cell contact, cytoskeletal, or even nuclear level that have evaded nanomechanical characterization due to their low mechanical stability will now be amenable to experimentation. More generally, and even beyond the mechanobiology field, our ability to measure protein dynamics over exceedingly long timescales using force as a denaturing probe enables us to directly capture rare, low-probability states that are not commonly observed in classically shorter experiments where the protein can only sample the major (un)folding barriers. Capturing transitions involving higher and more remote energy barriers in those proteins exhibiting a complex folding energy landscape might also have biological implications, for example capturing misfolded conformations closely related to the onset of aggregation diseases^59,60^ ^61–63^.

## Advantages and limitations

In contrast to AFM and optical tweezers, magnetic tweezers have only recently been implemented to study protein dynamics under force^37,38,40,55,64,65^. An inherent advantage of magnetic tweezers for measuring protein dynamics is its intrinsic force-clamp conditions, which allow for direct manipulation of the pulling force without requiring external force-feedback systems, hence enabling the equilibrium measurement. While the operational range of applied forces by magnetic tweezers is significantly narrower than that of AFM (0-120 pN vs. ~20-4,000 pN), it clearly outperforms AFM in its ability of manipulating forces in the low force regime (<20 pN) with sub-pN precision. Conversely, optical and magnetic tweezers largely overlap in the applicable range of forces; however, the inherent photodamage induced by the optical trap and intrinsic mechanical drift preclude optical tweezers from reaching the hallmark stability of our magnetic tweezers approach.

As is the case in any new-developing technique, single-molecule magnetic tweezers applied to protein dynamics has several directions for future instrumental improvements. From the technical perspective, the limited time resolution of magnetic tweezers is a drawback against the much faster AFM and optical tweezers instrumentation; although, the recent implementation of fast complementary metal–oxide–semiconductor (CMOS) cameras has increased the acquisition rate to the kHz range. Future improvements in image acquisition technology, combined with image processing methods—e.g. using parallelized algorithms, or incorporating dedicated units like FPGAs—could further push the current temporal limitation. Moreover, the enticing prospect of adding orthogonal fluorescence capabilities (successfully implemented in magnetic tweezers for DNA studies)^66,67^ might enable simultaneous force-light measurement, opening avenues to investigate, for instance, protein-protein interactions under force.

## Overview of the procedure

**Figure 1:**
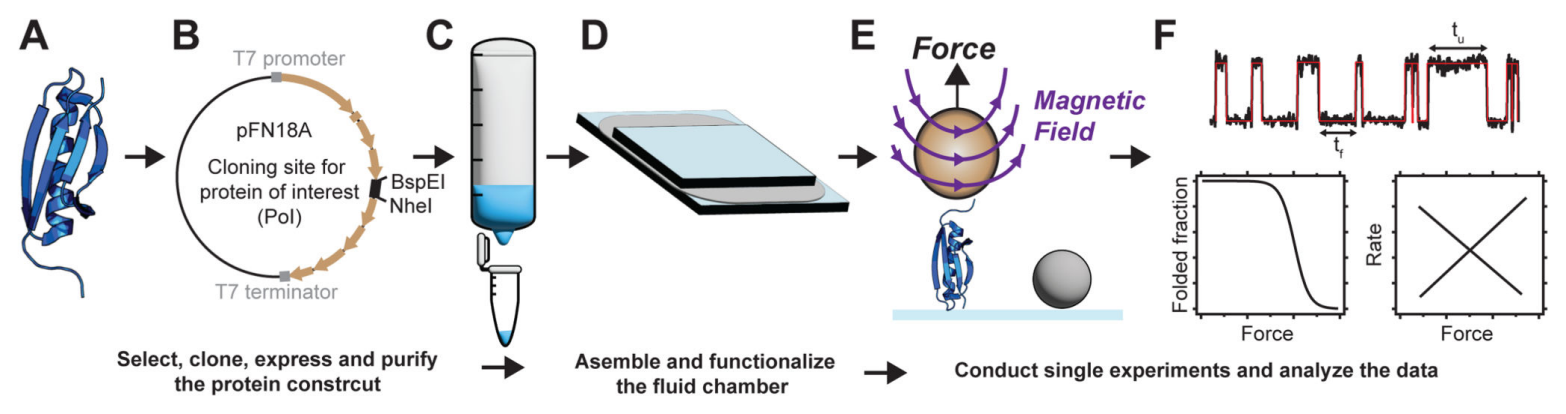
Schematics of the workflow to study the nanomechanics of an individual protein using single-molecule magnetic tweezers. **(A)** From the protein of interest (PoI), **(B)** clone the PoI gene into the expression-modified pFN18A plasmid. **(C)** Express and purify the protein construct. **(D)** Assemble and functionalize fluid chamber for specific and covalent anchoring of the protein construct. **(E)** Conduct nanomechanical experiments using the magnetic tweezers **(F)** Analyze and interpret the magnetic tweezers data.

Our protocol comprises three main stages (Fig. 1): *(i)* cloning, expression, and purification of the molecular construct containing the protein of interest; *(ii)* preparation and functionalization of the fluid chambers; *(iii)* conduction and analysis of the single-molecule experiments. We provide two alternative molecular strategies for cloning and tethering the protein of interest: *(i)* based on HaloTag and biotin anchors, suitable for proteins with a low mechanical stability (<65 pN); *(ii)* based on a double-covalent tethering using the SpyCatcher-SpyTag split protein technique, appropriate for proteins with a higher mechanical stability. This flexibility makes our protocol applicable to measure a broad array of proteins hallmarked by distinct mechanical stabilities—from labile focal adhesion^36,68^ proteins to stiff bacterial pilins^69^. Moreover, we highlight the importance of force calibration in single-molecule magnetic tweezers, detailing a procedure for instrumental calibration based on the elastic properties of unfolding proteins.

## Experimental design

### Single-molecule Magnetic tweezers

**Extended Figure 1:**
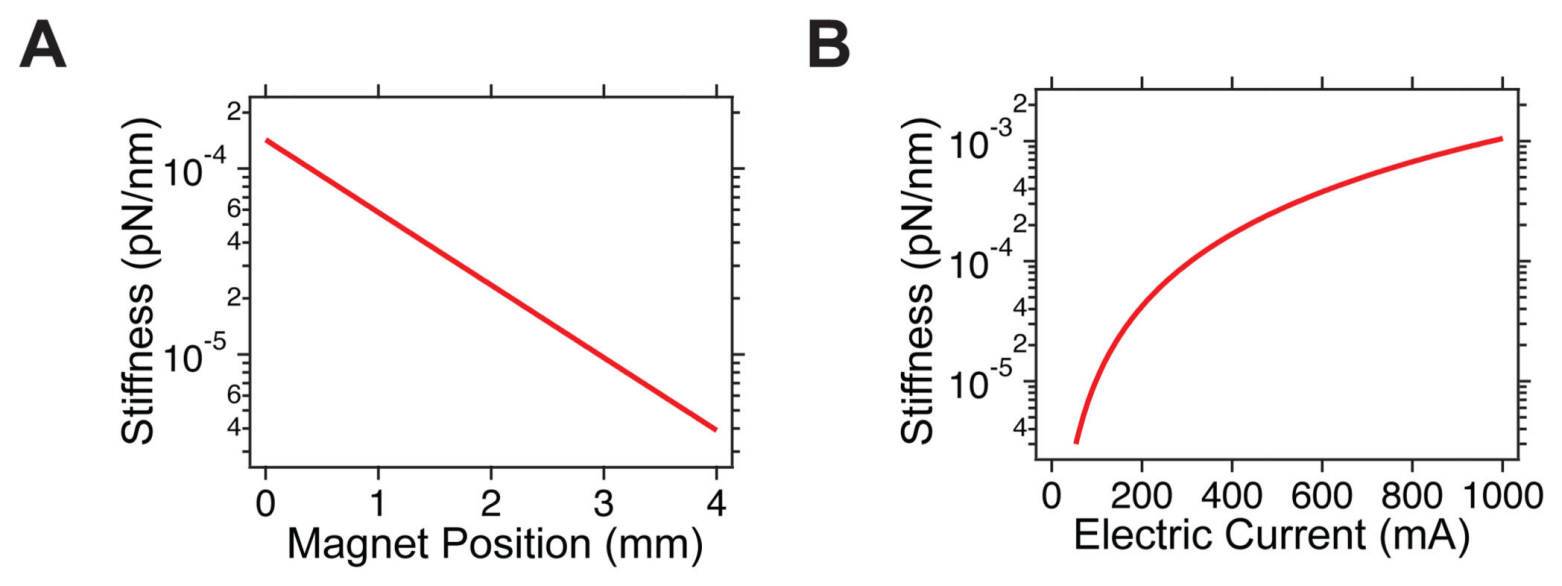
Stiffness of the magnetic trap created by the **(A)** N52 magnets (voice-coil configuration) and **(B)** magnetic tape head. The magnetic trap stiffnesses can be simply calculated as *dF/dz* being *z* the distance between the gap (magnets or tape head) and the magnetic bead. Due to the non-linearity of the *F*(*z*), the stiffness changes over the control parameter (magnet position or electric current), but in the operating regime the trap this results in a very soft trap (~10^-4^ pN/nm), resulting in effective force clamp conditions (no appreciable change in force over the range where the bead moves).

In magnetic tweezers, a magnetic field is used to apply calibrated forces to single molecules tethered between a functionalized superparamagnetic bead and a glass substrate, while their force-dependent conformational dynamics are measured by tracking the vertical position of the superparamagnetic bead with respect to a non-magnetic reference bead firmly attached to the glass substrate. Most magnetic tweezers configurations involve an inverted microscope to visualize the beads, using an objective mounted on a piezoelectric focus scanner to correct the focal position and infer the molecule’s relative extension. The magnetic field is applied above the fluid chamber using either a pair of permanent magnets or an electromagnet, which results in a pulling force acting on the superparamagnetic bead. By modifying the magnetic field intensity —which can be done by approaching or retracting the magnets or changing the electric current supplied to the electromagnet— the force can be exquisitely controlled and manipulated. The very different length scales over which the magnetic force changes (~10 µm) and the superparamagnetic bead vertically displaces upon an unfolding event (~20 nm) result in a very soft magnetic trap (~10^-4^ pN/nm, see Extended Fig. 1), implying that magnetic tweezers effectively provide intrinsic force-clamp conditions.

**Figure 2:**
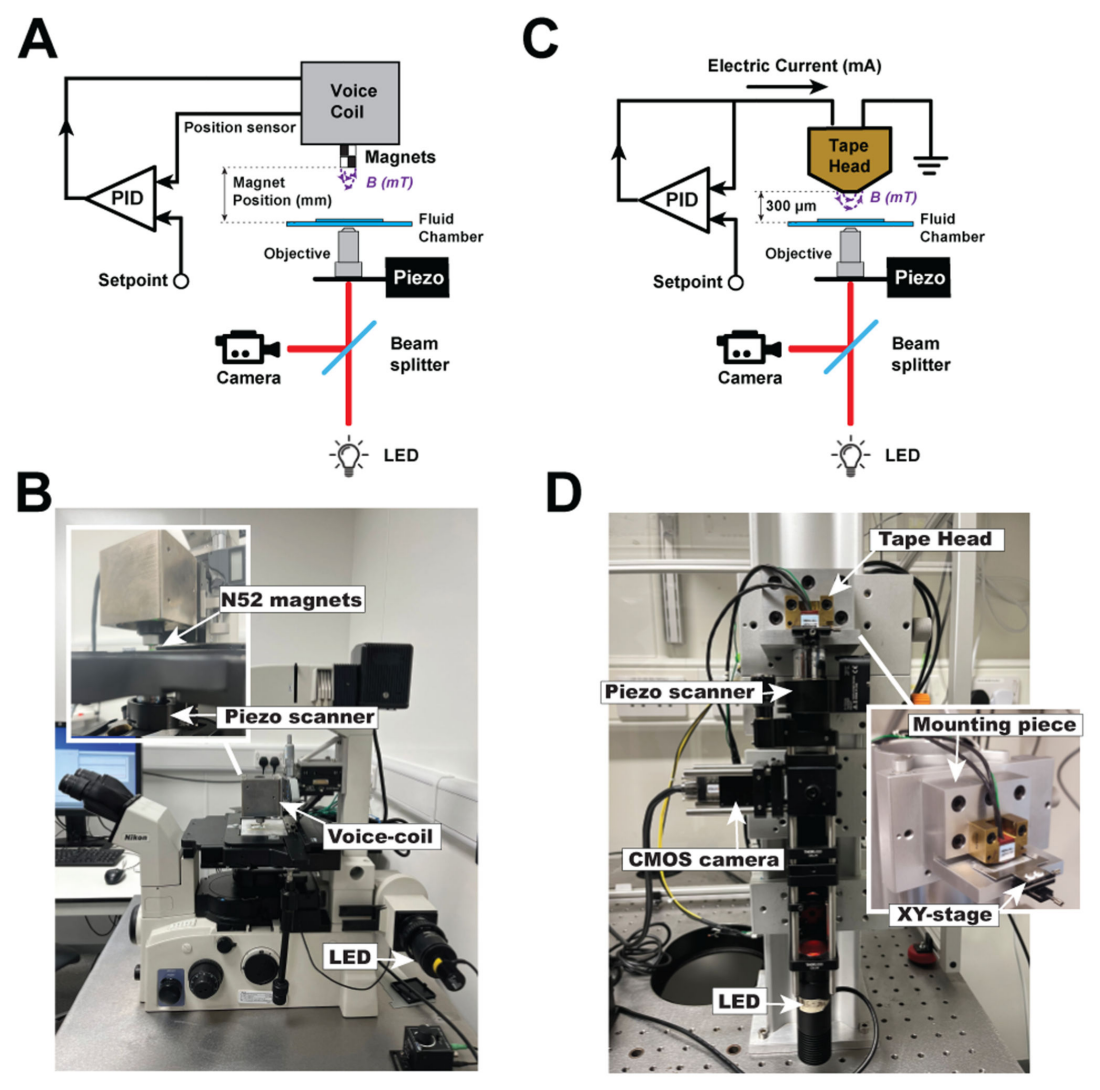
Overview of magnetic tweezers force spectroscopy. **(A)** Schematics of the magnetic tweezers instrument using the permanent magnet configuration. The magnets are placed on top of the fluid chamber, and their position is controlled by a voice coil maintained under feedback with a PID circuit. A commercial inverted microscope, with an objective mounted on a piezo focus scanner, allows tracking the vertical position of micron-sized beads. **(B)** Photograph of the instrument using permanent magnets, with labelling of some of its components. **(C)** Schematics of the magnetic tweezers instrument using the magnetic tape head configuration. The tape head is placed on top of the fluid chamber at a fixed distance of 300 µm and the magnetic field is applied by controlling the electric current supplied to the tape head using a PID circuit. The custom-made inverted microscope follows a similar arrangement as that used for the permanent magnet configuration. **(D)** Photograph of the magnetic tweezers instrument based on tape head configuration.

In our magnetic tweezers approach, we have implemented both the *(i)* permanent magnets and *(ii)* electromagnet strategies (Fig. 2) to create the magnetic field. *(i)* In our permanent magnet setup (Fig. 2 A, B), we employ a voice coil (LFA-2010, Equipment Solutions) to accurately position a pair of N52 magnets above the sample and displace them with micrometer resolution to change the force^37^. The voice coil is kept under electronic feedback with a custom proportional-integral-differential (PID) circuit, allowing us to displace the magnets with a ~10 Hz bandwidth and preventing their vertical drift while ensuring that the magnet position is at the desired setpoint. (ii) In our electromagnet configuration^51^ (Fig. 2C, D), we use a magnetic tape head (Dual Write head 902836, Brush Industries). By precisely positioning the tape head over the fluid chamber, we can control the pulling force by only manipulating the electric current using a current-clamp PID circuit to supply electric currents up to 1,000 mA, above which the head’s magnetization saturates. Compared to the voice-coil setup, the tape head configuration allows for very swift force changes, with a bandwidth over ~10,000 Hz.

### Image analysis algorithm

**Figure 3:**
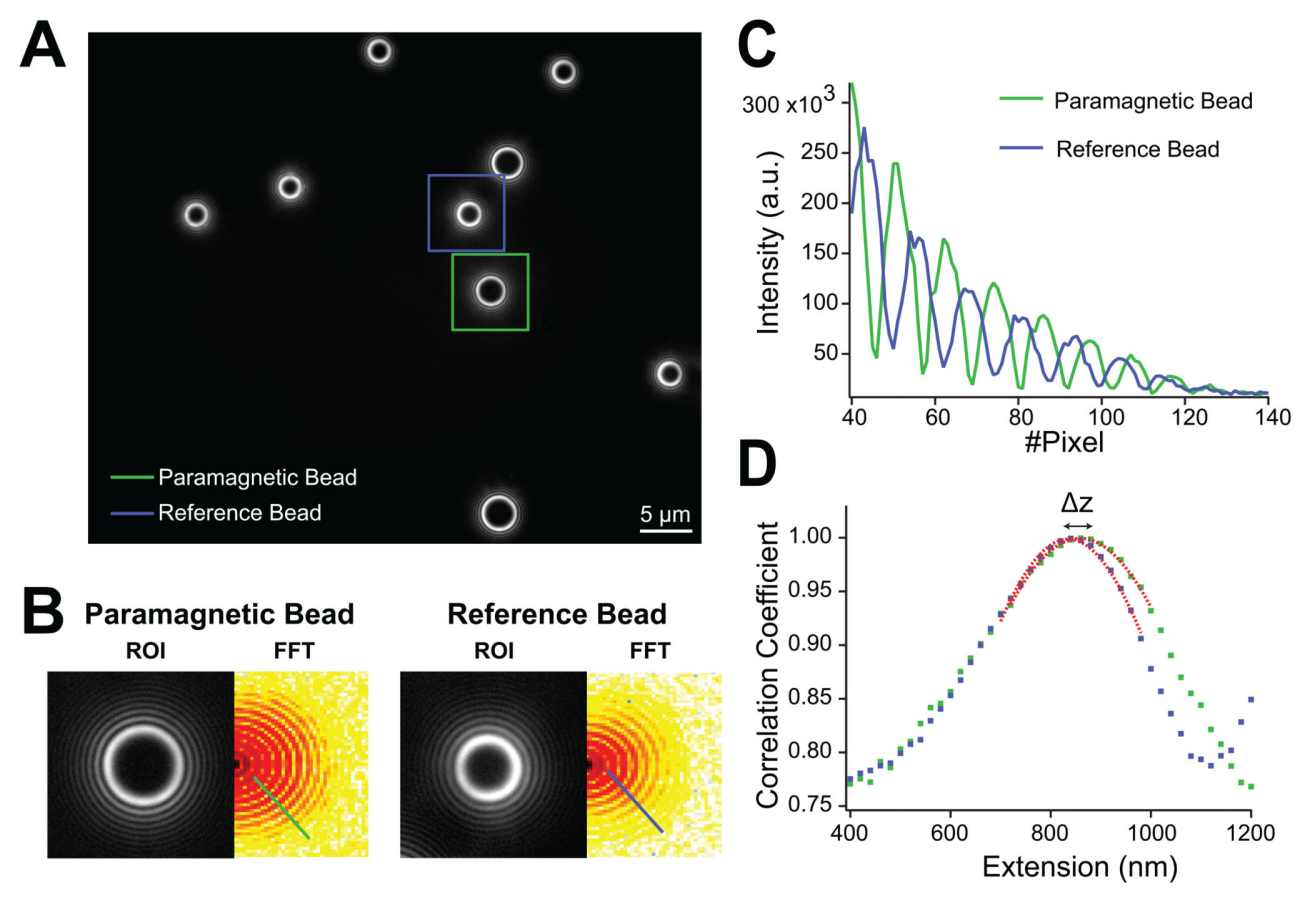
Image analysis algorithm **(A)** 60x image focusing on the bottom part of the fluid chamber displaying reference (smaller) and superparamagnetic (slightly bigger and brighter) beads (beads inside the blue and green square respectively). **(B)** The Fast Fourier Transformer (FFT) of the Region Of Interest (ROI) for the superparamagnetic and reference beads is calculated to integrate the pixel intensity at constant radial positions (green and blue lines), allowing **(C)** the calculation of the radial profiles, which are **(D)** correlated with a z-stack look-up library to obtain the bead height Δ*z* (molecular extension) as the difference in position of the Gaussian fits (red dotted lines) to the correlation profiles.

The conformational dynamics of the protein of interest in terms of its relative end-to-end length are inferred by tracking the relative vertical position of the superparamagnetic bead with respect to the non-magnetic reference bead attached to the surface using a standard image processing algorithm^37^. In our case, we use bottom illumination, meaning that we track the interference rings scattered by the superparamagnetic and reference beads. Upon identification of a pair of superparamagnetic-reference beads (Fig. 3A), the region of interest (ROI) that the camera will be tracking is established as the minimal rectangular region containing both beads. The time resolution of the experiment (frames-per-second) depends on the size of the ROI. The image from each bead is acquired as a 128 x 128 pixel square enclosing the bead and its interference rings (Fig. 3B). The radial intensity profile from these images is calculated in a two-step process, first computing the fast-Fourier transform (FFT) of the bead’s image and, second, the radial profile with a pixel-addressing algorithm that identifies the pixels with equal radial position and integrates their intensity (Fig. 3C; FKA algorithm^37^). At the start of the experiment, a *z*-stack look-up library of the beads’ profiles is built by running a piezo-scan, using 128 positions spaced by 20 nm, which gives a measuring range of 2.56 µm (SupplementaryVideo 1). Once the library has been built, the measuring process can start. Here, the real-time radial profiles from the magnetic and reference beads are correlated with all profiles in the stack library, and the vertical position of the superparamagnetic and reference beads is calculated by doing an analytical Gaussian fit (Caruana algorithm^70^) of the Pearson-correlation profile of each bead (Fig. 3D). The real-time relative extension of the bead is calculated as the difference in position between the Gaussian peaks, given that each profile is spaced by 20 nm. Due to this interpolation stage, we can achieve a spatial resolution larger than the piezo steps of 20 nm. When starting the measurement, the position of the reference bead is “locked” and every 1,000 frames, the objective position is corrected to bring the reference bead back to its initial locked position. This corrects for the local drift and allows for the hallmark long-term recordings of magnetic tweezers.

### Calibration of magnetic tweezers

The magnitude of the pulling force applied to a superparamagnetic bead depends on the bead’s properties (*i.e.* diameter, maximum magnetization, material, etc.) and the local magnetic field acting on the bead (intensity and gradient); in particular, $\vec{F}=\left(\vec{m}\cdot \vec{\nabla }\right)\vec{B}$, being $\vec{B}$ the magnetic field on the bead, $\vec{m}$ its magnetic moment (which also depends on $\vec{B}$). For all applications described here, we employ Dynabeads M-270^®^, which have a diameter of 2.8 µm and maximum mass magnetization of ~10.8 Am^2^/kg^71^. Changing the beads to ones with less (such as the smaller MyOne^®^) or more magnetic material (such as the larger Dynabeads M-450^®^) will respectively decrease or increase the maximum applicable force at a given magnetic field. The local intensity of the magnetic field can be changed by (i) approaching or retracting the pair of permanent magnets (closer magnets, higher field, higher force) or (ii) the electric current through the tape head. The field gradient is dominated by the width of the gap, either the tape head’s gap or the distance separating the two magnets. Due to the tape head’s much narrower gap than the distance between the two magnets (25 µm vs. ~150 µm), it can achieve similar forces despite having a lower maximum surface field (0.5 T vs. 2 T). Importantly, in both configurations, it is crucial to work right under the gap so that the only gradient direction is the vertical one, preventing lateral force components.

To calibrate our setups, we generate an empirical “force law” that relates the pulling force with the control parameter—the magnet position in the voice-coil configuration or the electric current in the tape head configuration. This approach requires an analytical expression relating the pulling force with the control parameter and the use of some molecular magnitude, the force-dependence of which is well described. By measuring such molecular magnitude as a function of the control parameter, we can estimate the fitting parameters that define our calibration curve. Developing an analytical expression for the magnetic field generated by a pair of permanent magnets is not trivial, as it depends on the magnet’s properties (geometry, surface field, etc.), which are generally hard to account for accurately. Instead, we employ an empirical expression that assumes that the magnetic force decays exponentially as a function of the magnet position (*MP*). Although more elaborate force vs. *MP* expressions have been proposed, for instance involving double exponential corrections^72^, our empirical law provides a good prediction of the applied force over the explored range (1-120 pN). In the case of the tape head, there is an analytical expression for the magnetic field generated by the tape head as a function of the distance and electric current (Karlqvist field)^73^; this allowed us to derive an analytical expression for the pulling force as a function of the electric current (at a given tape head-bead distance), which shows a quadratic dependence. Both expressions involve two fitting parameters (Eqs. 1 and 2).

(1) $$F_{m}\left(MP\right)=Ae^{-B\cdot MP},$$

(2) $$F_{m}\left(I\right)=AI^{2}+BI.$$

**Figure 4:**
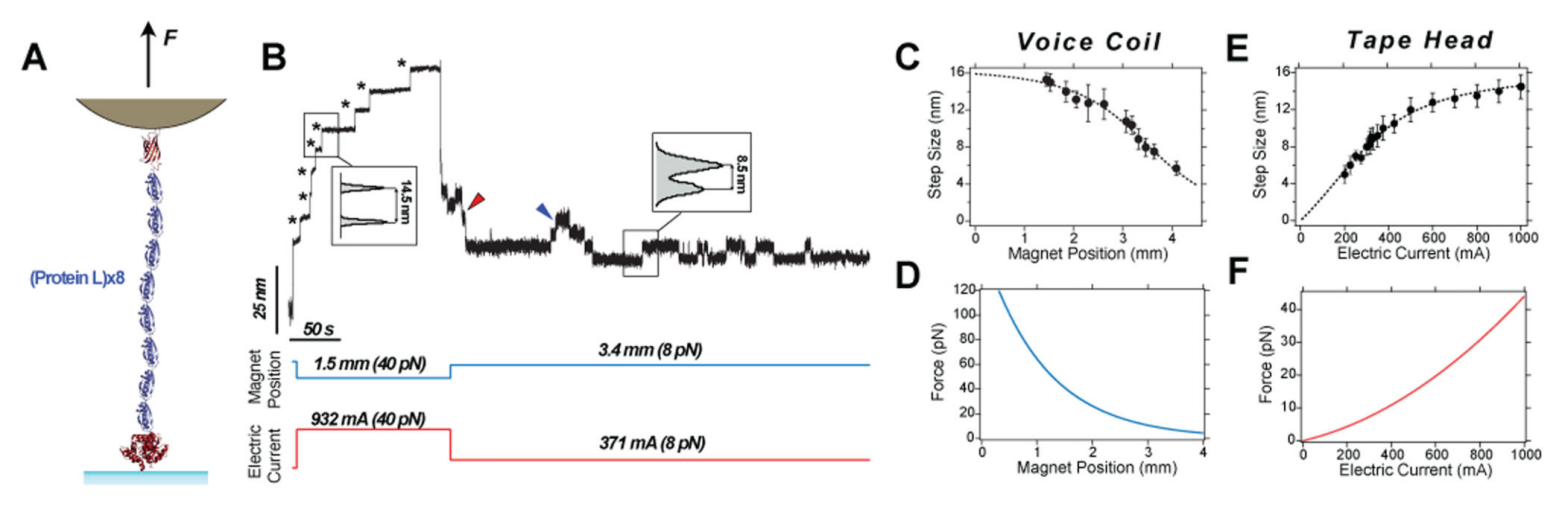
Step-size-based calibration of magnetic tweezers **(A)** A protein-L octamer is used as a molecular construct to derive a calibration law by correlating the sizes of the unfolding events with force. **(B)** Typical magnetic tweezers recording showing the dynamics of the protein L octamer under force. Upon a first high-force pulse (*F* = 40 pN—which corresponds to a magnet position of 1.5 mm in the voice-coil configuration (blue) or an electric current of 932 mA on the tape head setup (red)-, the eight protein domains readily unfold sequentially, showing a step-wise increase in the protein’s extension (stars). Subsequently, the force is lowered down to 8 pN (3.4 mm of magnet position (voice-coil) or 371 mA of electric current (tape head)), triggering the protein to refold, marked by a step-wise decrease in its extension (red arrow). Occasionally, a few unfolding events (blue arrow) are detected due to the competing unfolding and refolding kinetics at this force. The step-sizes scale with the applied force (~14.5 nm at 40 pN, and ~8.5 nm at 8 pN, insets) following polymer physics models of polymer elasticity such as the freely-jointed chain (FJC) model. The trace was smoothed with a 101-point 4th-order Savitzky-Golay algorithm. **(C)** Step sizes of protein L as a function of the magnet position for the voice coil configuration. Data from *n*=565 step sizes measured on *N>3* molecules. Error bars are SD. **(D)** Calibration law for the voice coil configuration using a pair of N52 magnets, providing the applied force as a function of the magnet position relationship. **(E)** Step sizes of protein L as a function of the electric current measured with the tape head configuration. Data from *n*=1,563 step sizes measured on *N>3* molecules. Error bars are SD. **(F)** Resulting calibration force law for the tape head configuration, relating the applied force with the electric current.

To determine the fitting parameters *A* and *B*, we use the *step sizes* characteristic of the unfolding and refolding of protein L under constant force^37,74^ as a model molecular quantity, given that their force dependence is well described (Fig. 4A). When a protein unfolds under force, it becomes an unstructured polymer that equilibrates at an average end-to-end extension, according to standard polymer physics elasticity models such as the freely jointed chain (FJC) model (Fig. 4B). In the case of protein L, the force dependence of its step sizes as a function of force can be described as:

(3) $$\langle \Delta z\rangle \left(F\right)=\Delta L_{C}\left[\coth\left(\frac{Fl_{K}}{kT}\right)-\frac{kT}{Fl_{K}}\right],$$

where *⊿L*_C_=16.3 nm and *l_K_*=1.1 nm^75^, and *kT*=4.11 pN nm. Therefore, measuring <*⊿z*> as a function of the magnet position *MP* (for the voice-coil configuration) or of the electric current *I* (for the tape head configuration) enables the estimation of the calibration parameters *A* and *B*. When using a pair of N52 magnets and M-270 superparamagnetic beads, Eq. 1 takes the form:

(4) $$F_{m}\left(MP\right)=158.4e^{-0.9\cdot MP},$$

where *[F]*=pN and *[MP]*=mm, and therefore *[A]*=pN and *[B]*=mm^*-1*^. Given that the range of the voice coil in our configuration (LFA-10 actuator, Equipment solutions) is ~8 mm and that the maximum distance that the magnets can be approached is ~0.3 mm (due to the thickness of the fluid chamber), the range of forces in the voice coil configuration is 0.1-121 pN.

**Extended Figure 2:**
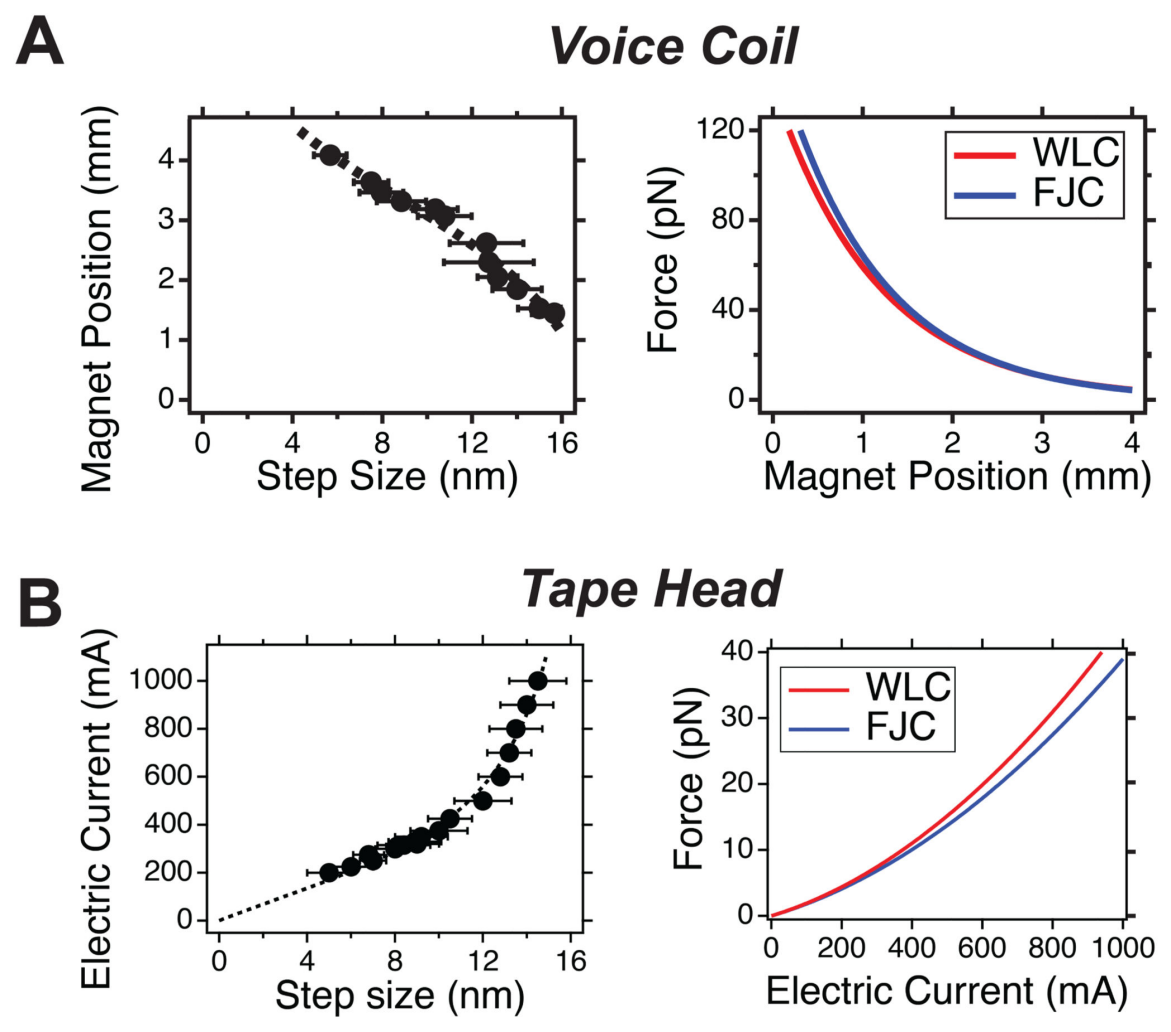
Calibration of the **(A)** voice coil-based or **(B)** tape head-based magnetic tweezers using the worm-like chain model for polymer elasticity (left) and comparison of the calibration using the WLC and FJC (right). The FJC gives a lower contour length (⊿*L*_c_=16.3 nm) compared to the WLC (⊿*L*_c_=18.6 nm). All error bars are SD.

For the case of the tape head, when using a Brush Industries Dual Write 902836 tape head placed at a distance of 300 µm from the magnetic beads and M-270 superparamagnetic beads, Eq. 2 becomes:

(5) $$F_{m}\left(I\right)=\left(2.786\times 10^{-5}\right)I^{2}+\left(1.640\times 10^{-2}\right)I,$$

where *[F]*=pN and *[I]*=mA,and therefore [A]=pN mA^-2^ and [B]= pN mA^-1^. Given that the tape head saturates at electric currents of ~1,000 mA, the range of forces in the tape head configuration is 0-44 pN. The resulting calibration curves in each case are shown in Fig. 4 C, D and Fig. 4 E, F. While in magnetic tweezers using the FJC model is the natural choice given its *x*(*F*) form, the worm-like chain (WLC) model can be alternatively used, which gives rise to an equivalent calibration curve (Extended Fig. 2).

### Design of protein constructs

In our magnetic tweezers approach, we employ HaloTag chemistry to covalently anchor the N-terminus of the protein construct to a glass cover slide, while the C-terminus attaches to the superparamagnetic bead, either with a biotin-streptavidin interaction or also by means of HaloTag chemistry (see below). The HaloTag is a derivatized bacterial hydrolase enzyme containing a haloalkane dehalogenase that reacts with high specificity with chloroalkane groups—in our approach, we use the HaloTag amine (O4) ligand—forming a covalent bond by a nucleophilic displacement of the terminal chlorine from the HaloTag ligand^49^. To allow covalent anchoring of Halotagged-protein constructs, the magnetic tweezers experiments are conducted on custom-made fluid chambers that have been previously functionalized to harbor the HaloTag ligand. The protocol for producing the fluid chambers is detailed in the next section.

**Figure 5:**
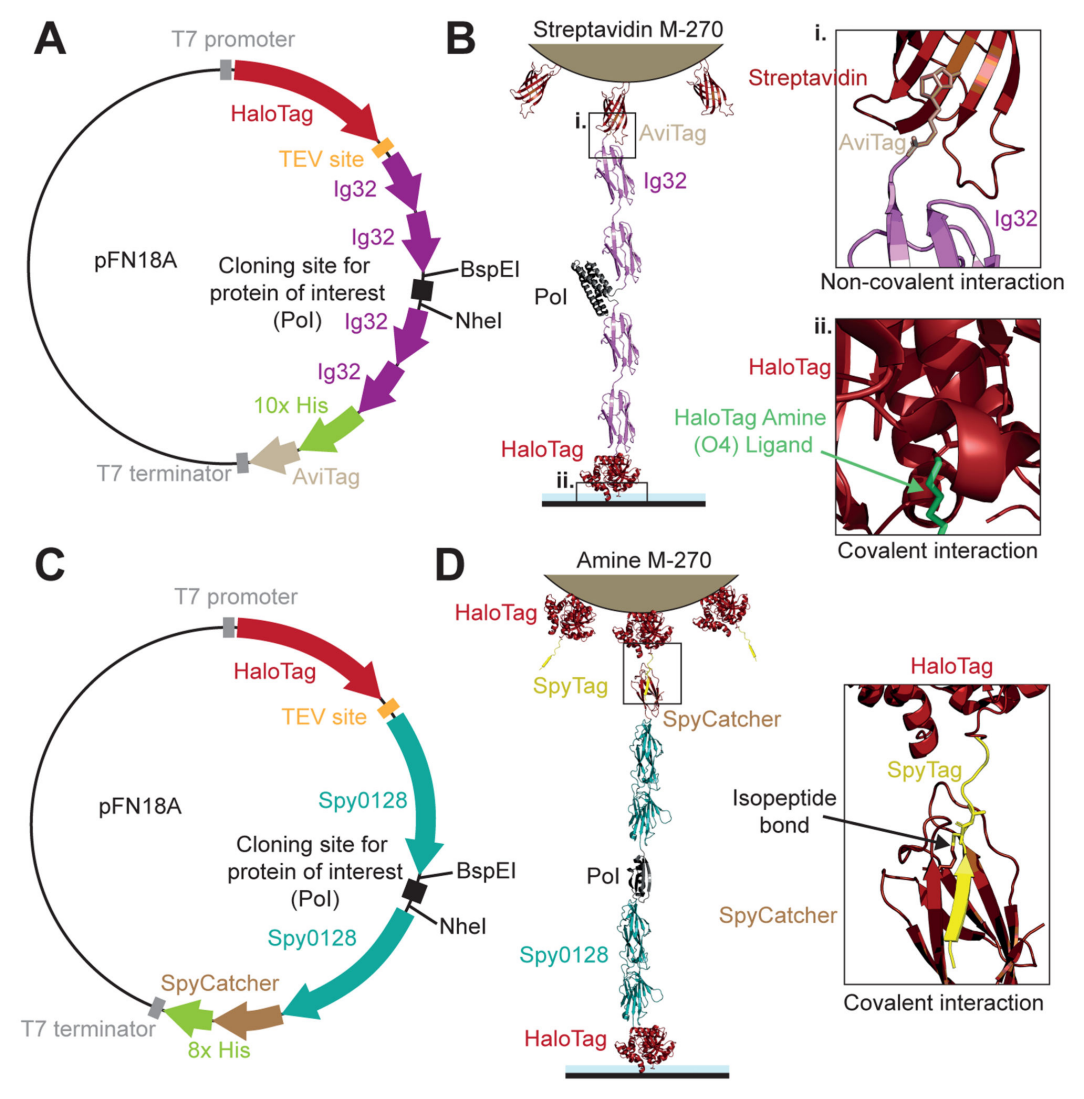
Protein construct designs and click-chemistry strategies. **(A)** Schematics of the modified pFN18A expression plasmid used to engineer proteins to be stretched in magnetic tweezers experiments involving an N-terminal HaloTag and a C-terminal AviTag anchoring. Between the four Ig32 domains (stiff molecular handles), the BspEI and NheI restriction sites used to routinely insert the protein(s) of interest. The HisTag is added for purification purposes. **(B)** Schematics of the resulting protein construct, here, using a talin R3^IVVI^ monomer as the protein of interest (PoI), immobilized between a streptavidin-coated superparamagnetic bead and a functionalized cover slide. Inset **i.** shows the non-covalent interaction between the C-terminal AviTag and streptavidin from the superparamagnetic bead. Inset **ii.** shows the covalent attachment between the N-terminal HaloTag and the HaloTag Amine (O4) Ligand. **(C)** Schematics of the pFN18A expression plasmid used to engineer those proteins requiring an N-terminal HaloTag and a C-terminal SpyCatcher. Between the two inextensible Spy0128 domains (stiffer molecular handles), the BspEI and NheI restriction sites enable insertion of the protein of interest. **(D)** Schematics of the resulting protein construct, here using a protein L monomer as the PoI. The protein construct is covalently immobilized to the glass cover slide using the N-terminal HaloTag. The C-terminal SpyCatcher covalently binds to the SpyTag, attached to the functionalized amine superparamagnetic bead. The inset shows the intermolecular isopetide bond enabling the covalent click-chemistry reactivity between the SpyCatcher and the SpyTag. Plasmid sequences have been uploaded to Addgene (pFN18A-HaloTag-Biotin: Addgene plasmid #206039; pFN18A-HaloTag-SpyCatcher Addgene plasmid #206041).

The protein domain(s) to be studied using magnetic tweezers are inserted into a larger protein construct to *1)* allow chemical anchoring to the surface and to the superparamagnetic bead; *2)* spatially separate the protein of interest from the bead and surface to prevent artefactual interactions. We employ two different protein constructs depending on the mechanical properties of the protein of interest. ***i)*** For mechanically weak proteins (unfolding forces <65 pN), the protein of interest is inserted between two Ig32 titin domains, with the HaloTag at the N-terminus and an AviTag for biotinylation at the C-terminus (Figure 5 A,B). The mechanically stiff titin Ig32 domains serve as molecular spacers and lack any mechanical response at forces <100 pN^52^. With this protein construct, we employ commercially available streptavidin-coated superparamagnetic beads (M-270 Dynabeads^®^, Thermofisher). At forces <10 pN, the lifetime of this tether is >10^6^ s ^38^. ***ii)*** When studying mechanically stiff proteins (*i.e.* unfolding forces high enough to compete with the unbinding of the biotin-streptavidin non-covalent bond), a double covalent strategy also involving HaloTag conjugation with the superparamagnetic bead can be employed (Figure 5 C,D). In this case, the protein of interest is flanked by two Spy0128 domains (which are mechanically inextensible due to an intramolecular isopeptide bond^76^), an N-terminus HaloTag, and a C-terminus SpyCatcher. By functionalizing M-270^®^ amine superparamagnetic beads with HaloTag-SpyTag protein (see below), the protein of interest can be covalently conjugated with the superparamagnetic beads taking advantage of the SpyTag-SpyCatcher split-protein technique (i.e., forming an intermolecular isopeptide bond^50^), allowing for a double covalent tether capable of withstanding large mechanical forces without detachment. Figure 5 A,C shows a schematic of the modified pFN18a plasmid for each construct, indicating the BspEI and NheI restriction sites used in this case for insertion of the protein of interest. Figure 5 B,D shows schematics of the resulting protein constructs tethered between a functionalized surface and a superparamagnetic bead, with special emphasis on the different chemical reactivity involved in anchoring the protein constructs.

## Production of fluid chambers for magnetic tweezer experiments

### Reagents

- T7 Express Competent *E. coli* (High Efficiency) cells (New England BioLabs). Genotype: fhuA2 lacZ::T7 gene1 [lon] ompT gal sulA11 R(mcr-73::miniTn10--TetS)2 [dcm] R(zgb-210::Tn10--TetS) endA1 Δ(mcrC-mrr)114::IS10.
- LB broth (Miller) powder microbial growth medium Luria Broth, Sigma-Aldrich.
- Ampicillin sodium salt (ThermoFisher Scientific).
- Chloroamphenicol (Sigma-Aldrich).
- Isopropyl β-D-1-thiogalactopyranoside (IPTG), (Cambridge Biosciences).
- Lysis buffer pH 7.5 [20 mM HEPES, 300 mM NaCl, complete protease inhibitor cocktail EDTA-free tablets Roche (Sigma-Aldrich), 0.8 mg/mL of lysozyme, 0.8 μg/mL of DNase (Deoxyribonuclase I from bovine pancreas, Sigma-Aldrich), 0.8 μg/mL of RNase (RNase a molecular biology grade, Apollo Scientific), 4 mM phenylmethylsulfonyl fluoride (Sigma-Aldrich) and 10 mM MgCl_2_].
- Imidazole, ≥99.8% (Santa Cruz Biotechnology)
- SnakeSkin Dialysis Tubing (ThermoFisher Scientific).
- MiliQ water, >18 MOhm-cm, 0.22 *μ*m filtered, ultrapure (type 1) water
- Hellmanex III soap (Hellma Analytics)
- Acetone CHROMASOLV, ≥99.8% (Fisher scientific)
- Methanol anhydrous, 99.8% (Sigma-Aldrich)
- 3-aminopropyl)trimethyloxysilane, 97% (Sigma-Aldrich)
- PlusOne Repel-Silane ES, 2 % dimethyldichlorosilane in octamethylcyclooctasilane (Cytiva)
- PBS, 50 mM sodium phosphate (Na_2_HPO_4_ and NaH_2_PO_4_) 150 mM NaCl pH 7.3 (Sigma-Aldrich)
- L-Ascorbic acid, 99 % (Sigma-Aldrich)
- Albumin – Sulfhydryl Blocked, ≥98 % (Virion-Serion)
- HaloTag Amine (O4) Ligand (Promega)
- Glutaraldehyde solution, Grade I, 25 % in H2O (Sigma-Aldrich)
- Amino polystyrene beads, 2.68 µm, 5.0 % w/v (Spherotech)
- Dynabeads M-270 streptavidin, 2.8 µm, 10 mg mL^-1^ (ThermoFisher Scientific)
- Dynabeads M-270 Amine, 2.8 µm, 10 mg mL^-1^ (ThermoFisher Scientific)
- Silicone Oil, viscosity 10,000 cSt at 25°C (Sigma-Aldrich)

### Equipment

- Beckham Avanti J-26 XP centrifuge (Beckman Coulter Life Sciences)
- HTU DIGI-French-Press (G. Heinemann Ultraschall- und Labortechnik).
- Cell strainer: Corning Falcon Cell Strainer for use with 50 mL conical tubes pore size 40 µm, blue Nylon, sterile (Sigma-Aldrich).
- HisTrap HP His tag protein purification columns, 1 mL (Cytiva).
- Äkta purifier (GE Healthcare/Amersham Biosciences).
- 4-15 % mini-PROTEAN TGX Precast protein gels (Bio-Rad)
- Mini-PROTEAN Tetra Vertical Electroforesis cell (Bio-Rad)
- Vivaspin centrifugal device: Vivaspin 6, 10000 MWCO PES (Sartorius)
- Superdex 200 Increase small-scale SEC columns (cytiva)
- 10/300 GL column (Cytiva)
- Glass staining jars for 22 x 22 mm cover slides, 8 mL volume (Agar scientific)
- Glass staining jars for 26 x 70 mm cover slides, 55 mL volume (Fisher Scientific)
- Alumina coverglass staining rack, 90° V-through with an 8 mm open slot at the bottom suitable for plasma cleaners (ProSciTech)
- Clifton Heated Timed Ultrasonic Bath, 1 L (Fisher Scientific)
- Tweezers
- Basic Plasma Cleaner (used with air) PDC-32G-2 (Harrick Plasma)
- Bemis Parafilm M Laboratory Wrapping film (Fisher Scientific)
- 22 x 22 mm cover slide, 1.5 thickness, Class 1 glass (Avantor), ¨*tops*¨
- 24 x 40 mm cover slide, 1.5 thickness, Class 1 glass (Avantor), ¨*bottoms*¨
- Standard incubator with natural convection, 20 L capacity (Binder)
- Stirring Hot Plate, Ceramic, (Scientific Laboratory Supplies)
- IKA Vortex 3 mixer, (Imlab)
- Home-made Humidity chamber
- Loopster basic digital (Imlab)
- Sigma 1-14K refrigerated microcentrifuge (Sigma Laborzentrifugen GmbH)
- Laser cutter (VLS3.50, Universal Laser systems) - optional.

## Procedure

**Critical** All buffers must be filtered before use.

**Caution** Avoid direct contact with reagents and equipment. Use appropriate personal protective equipment (laboratory coat, gloves and protective goggles) when indicated.

### Design and expression of protein constructs

*Cloning and expression of [HaloTag-(Ig32)_2_-(Protein of Interest)-(Ig32)_2_-10xHisTag-AviTag] protein constructs (Timing: 6 days)*

1. Using GeneArt (Thermo Fisher Scientific), design a Protein of Interest (PoI) gene sequence flanked by the Kpn21 and NheI restrictions sites and insert it into the modified pFN18a plasmid vector (Fig. 5A)
2. Transform the modified pFN18a plasmids into T7 competent cells, carrying a pBirAcm plasmid (Avidity) for the *in vivo* biotinylation of the protein constructs
3. Grow T7 competent cells in Luria-Bertani (LB) medium supplemented with 100 μg/mL of ampicillin and 34 μg/mL Chloramphenicol at 37 °C
4. When cells reach an optical density of ~0.6 (at 600 nm), induce the overexpression of the protein construct with 1 mM of isopropyl β-D-1-thiogalactopyranoside supplemented with 50 μM of biotin (Invitrogen) and let them overexpress the protein constructs for 16 h at 20 °C

*Cloning and expression of [HaloTag-Spy0128-(Protein of Interest)-Spy0128-SpyCatcher-8xHisTag] protein constructs (Timing: 6 days)*

- Using GeneArt (Thermo Fisher Scientific), design a Protein of Interest (PoI) gene sequence flanked by the Kpn21 and NheI restrictions sites and insert it into the modified pFN18a plasmid vector (Fig. 5C)
- Transform the modified plasmids into T7 competent cells.
- Grow T7 competent cells in LB medium supplemented with 100 μg/mL of ampicillin at 37 °C
- When cells reach an optical density of ~0.6 (at 600 nm), induce the overexpression of the protein construct with 1 mM of IPTG and let them overexpress the protein constructs for 16 h at 20 °C

### Purification of protein constructs (timing: 2 days)

The purification of [HaloTag-(Ig32)_2_-PoI-(Ig32)_2_-10xHisTag-AviTag] and [HaloTag-Spy0128-PoI-Spy0128-SpyCatcher-8xHisTag] protein constructs follows the exact same protocol.

- After induction, centrifugate the cells (500 mL) for 20 min at 3000g
- Resuspend the bacterial pellet in 25 mL of lysis buffer
- Incubate the cells on ice for 30 min with the lysis buffer
- After incubation, disrupt the cells using a French press
- Clear the lysate by centrifuging it for 45 min at 39000g
- Collect the supernatant and filter it with a cell strainer (40 μm filter)
- Equilibrate the 1 mL HisTrap HP column with buffer A (containing 20 mM HEPES pH 7.5 and 300 mM NaCl)
- Load the clarified lysate onto the column using a pump
- Wash the column with Buffer A supplemented with 10 mM of imidazole using an Äkta Purifier until the absorbance reaches the baseline (usually 100 Column Volume, CV)
- Elute the protein using a linear gradient of Elution buffer (buffer A supplemented with 500 mM of imidazole), over 40 CV
- Collect the eluted fractions and analyze them by SDS-PAGE
- Dyalize overnight the collected fractions against a buffer containing 20 mM HEPES pH 7.5 and 150 mM NaCl
- Contrate the protein sample using a Vivaspin centrifugal device
- Purify the concentrated protein sample with a Superdex 200 increase 10/300 GL column using an Äkta Purifier and buffer A supplemented with 10% of glycerol as a running buffer
- Analyze the eluted fractions by SDS-PAGE, and flash-freeze and store the pure protein constructs at -80ºC

### Assembly and preparation of the fluid chambers

*Cleaning the bottom cover slides (timing: 3 hours)*

- Using a pair of tweezers, place the bottom cover slides (24 x 40 mm) into the staining jars. To maximize the space in the staining jar, the cover slides can be placed in *zig-zag* configuration
- Fill the staining jars with a 1 % Hellmanex III solution and place them into a sonicator for 30 min at 50ºC
- After sonication, thoroughly rinse the staining jars with double distilled water (x10)
- Fill the staining jars with acetone and sonicate the cover slides for 30 min at room temperature. This cleaning step must be performed inside a fume hood
- Discard the acetone into an appropriate organic waste disposal container
- Rinse thoroughly with double distilled water (x10)
- Repeat a sonication cycle, this time with methanol (or ethanol) for 30 min
- Discard the methanol in an appropriate organic waste disposal container
- Place the cover slides in a temperature-resistant cover slide holder
- Gently dry the methanol excess with compressed air
- Place the cover slides in an oven for 40 min at 100 °C

**Critical step:** At this point, the cover slides are cleaned and ready for silanization. The cleaned cover slides can be readily silanized (step 12) or, if preparing a big batch of cleaned cover slides, stored in a desiccator under vacuum for several months.

*Silanization of the bottom cover slides (timing: 2 hours)*

- Place the cleaned cover slides in a holder and activate them using an air plasma cleaner for 20 min at the highest intensity
- Transfer the cover slides to the staining jars and fill them with a 0.1% solution of (3-aminopropyl)trimethyoxysilane in methanol (or ethanol)
- Incubate for 30 min inside a fume hood
- Discard the amino-silane solution from the staining jars and rinse the cover slides x2 with methanol (or ethanol)
- Place the cover slides in a temperature-resistant holder and gently dry them with compressed air
- Dry the cover slides for >1 hour in the oven at 100 °C
- The silanized bottom cover slides can be used straight away for the assembly step or stored in a desiccator under vacuum for ~2 months. Also, store the opened amino-silane bottle in the desiccator for up to 1 month

*Cleaning and silanization of the top cover slides (timing:1 h 45 min)*

- Place the top cover slides (22 x 22 mm) in the staining jars
- Sonicate the cover slides for 30 min with a 1% Hellmanex III solution at 50ºC
- Discard the solution and wash the cover slides thoroughly with double distilled water (x10)
- Sonicate the cover slides for 10 min with methanol (or ethanol)
- Appropriately discard the methanol solution and fill the staining jars with repel-silane
- Incubate the cover slides with the repel-silane for 30 min inside a fume hood
- Appropriately discard the silane solution and wash the cover slides twice with methanol
- Place the cover slides in a temperature-resistant holder and gently dry them with compressed air
- Dry the cover slides in the oven at 100 °C for 30 min
- Store the cover slides in a desiccator under vacuum. This hydrophobic treatment can last for up to ~3 months

*Assembly of the fluid chambers (timing: 15 min)*

**Critical** How to assemble fluid chambers (steps 29 to 32) is illustrated step by step in Supplementary Video 2.

**Figure 6:**
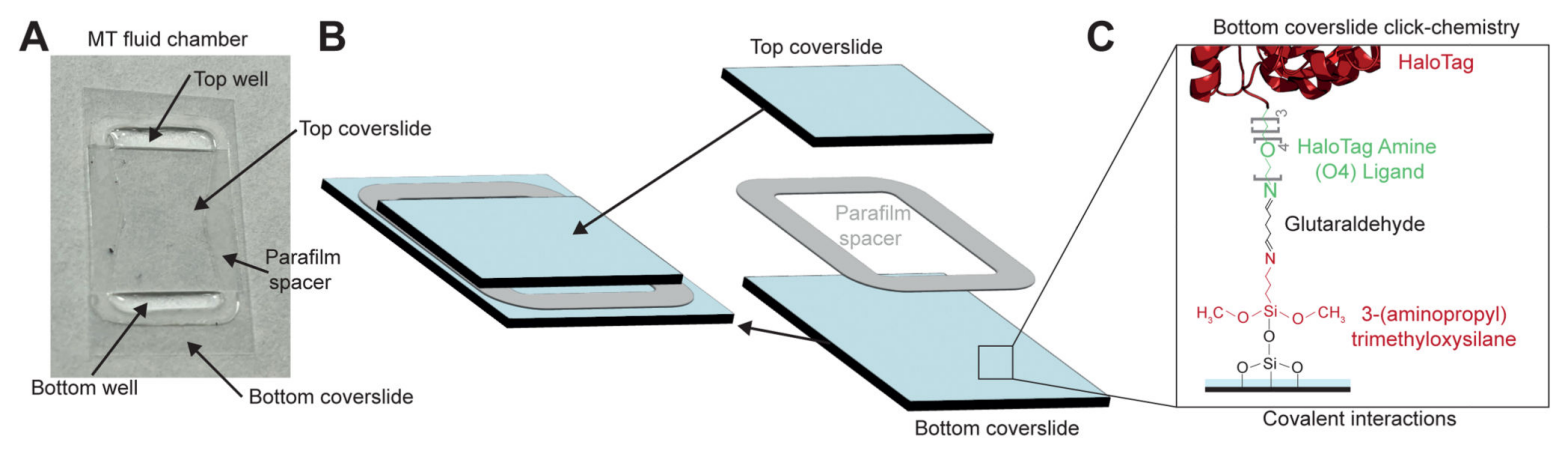
Fluid chamber configuration and assembly. (**A**) Photograph of a fluid chamber employed in our magnetic tweezers experiments. (**B**) Schematics of the assembly of a fluid chamber. **(C)** Surface chemistry employed in the functionalization of the bottom cover slide with the HaloTag O4 ligand.

- Preheat a hot plate to 100 °C
- Assemble the fluid chamber by placing the parafilm spacer on top of the silanized bottom cover slide and the hydrophobic top cover slide in the middle of the bottom glass, so that the parafilm pattern creates two similarly sized wells (Fig. 6). Assemble as many chambers as desired; once prepared and functionalized, they can be stored for ~1 month if kept hydrated by periodically topping up the wells with the working buffer
  **Critical Step:** The parafilm spacers (Fig. 6B) can be cut using a laser cutter (red color 35 % power, 20 % speed, PPI 400 and 4.5 mm z-axis). If no laser cutter is available, the parafilm spacers can be cut manually using a regular cutter.
- Place the chambers on the hot plate, gently pressing with the tweezers on the top cover slide until the parafilm melts and becomes transparent. If the parafilm on the edges bends over, gently press it towards the bottom cover slide until it sticks to it. Repeat the process with all the assembled chambers
- Place the assembled chambers in the humidity chamber. This can be a large plastic Petri dish with wet paper and a parafilm sheet on top. When closed, this creates a humid environment that will prevent evaporation

*Bottom cover slide HaloTag ligand hybridization (timing: 16 hours)*

- For all wet-chemistry functionalization steps, the chambers should be kept in the humidity chamber, which is tilted to facilitate the flow through when exchanging buffers.
- Prepare a 1% glutaraldehyde solution in PBS (*e.g.,* 54 µl of the 25% glutaraldehyde solution to 1.3 mL of PBS). Add ~50 µl of the glutaraldehyde solution on the top well, checking that the solution flows through the fluid channel, and another ~50 µl on the bottom well to ensure that the solution can flow between wells
- Bring the humidity chamber back to a flat surface and top up the wells of the fluid chamber. There should be a visible amount of fluid without overflowing the top cover slide. Incubate for 60 min at room temperature

**Critical step** Use a Grade I glutaraldehyde solution (25% in H_2_O) sold in 1 mL ampoules, and store them in 60 µl aliquots in a -20ºC freezer

- Prepare a ~0.03% w/v solution of polystyrene 2.68 µm beads in PBS (*e.g.* 9 µl of beads to 1.3 mL of PBS)
- Tilt the chambers and discard the glutaraldehyde solution by pipetting it out from the bottom well

**Troubleshooting**

- Add ~50 µl of the polystyrene bead solution to the top well and discard the solution from the bottom well. Add another ~50 µl and repeat to ensure that no solution from the previous step remains in the fluid chamber
- Add ~50 µl to the top well and bring the humidity chamber back to a flat position. Top up the wells and incubate for ~20 min at room temperature
- Prepare a 20 µg/mL solution of HaloTag Amine (O4) ligand in PBS (5 µl of the stock ligand solution to 1.3 mL of PBS)

**Critical Step**: The HaloTag Amine (O4) Ligand comes as a powder that needs to be dissolved in dimethyl sulfoxide (DMSO) as described by the manufacturer (1 mL DMSO added to the 5 mg of ligand). To ensure a longer shelf lifetime, it is advisable to aliquot it in small volumes and store it in an inert atmosphere (*i.e*., argon) at -80 °C. The frozen aliquots can be used for up to~12 months.

- Discard the polystyrene beads solution by tilting the chambers and removing the buffer from the lower well. Thoroughly wash the chambers three times by circulating PBS. This is important to remove non-attached beads or any remaining glutaraldehyde that might compete with the HaloTagligand
- Add ~50 µl of the HaloTag ligand solution to the top well and discard the solution from the bottom well. Repeat. Add ~50 µl to the top well and bring the humidity chamber back to a flat position. Top up the wells and incubate overnight at room temperature
- Prepare the passivation buffer (20 mM Tris-HCl pH 7.4, 150 mM NaCl, 2 mM MgCl_2_, 1% w/v sulfhydryl-blocked BSA, 0.01% sodium azide)

**Criticalstep**: The passivation buffer can be prepared in advance and stored in aliquots at -20ºC.

- Put the humidity chamber in a tilted position and discard the HaloTag ligand solution from the bottom well of the fluid chambers
- Wash the fluid chamber four times with ~100 µl of the passivation buffer
- Bring the humidity chamber to a flat position and incubate for at least three hours at room temperature.
- After three hours, the fluid chambers are ready to use

**Troubleshooting**

*HaloTag ligand hybridization to amine-terminal superparamagnetic beads (only for double covalent assemblies) (timing: 2 days)*

Steps 71 to 85 are only necessary if using the double covalent strategy, which involves HaloTag anchoring to both the bottom cover slide and the superparamagnetic bead. If using streptavidin/biotin to bind the protein construct to the superparamagnetic bead, please proceed to step 86.

- Take 10 µl of M-270 amine beads and resuspend them in 430 µl of PBS
- Centrifuge the beads at 15,000 rpm for ~2 min to sediment them and discard the supernatant
- Resuspend the beads in 430 µl of 1% glutaraldehyde solution in PBS

**Critical step** Do not use the vortex mixer as it could damage the beads.

- Leave the beads in the glutaraldehyde solution for one hour under ~18 r.p.m. rotation (to avoid clustering) at room temperature
- Wash the beads 5 times with PBS by spinning them down and resuspending in PBS
- After discarding the supernatant for the fifth time, energetically resuspend the beads in 430 µl of a 20 µg/mL solution of HaloTag Amine (O4) Ligand in PBS
- Leave the beads incubating with the HaloTag ligand overnight at room temperature under rotation
- After incubation, wash the beads twice with PBS
- When discarding the supernatant for the second time, incubate the beads in 430 µl of passivation buffer and incubate under rotation at room temperature for at least 4 hours.
- The beads can be stored on the passivation buffer under rotation in a fridge for ~2 weeks
- Take 20 µl of the O4 ligand derivatized M-270 amine beads and dilute them in 180 µl of PBS
- Wash the beads by spinning down the solution at 15,000 rpm and resuspend them in 200 µl of PBS
- Discard the supernatant and incubate the derivatized M-270 amine beads in 10 µl of a 20 µM HaloTag-SpyTag dilution for 30 minutes at room temperature under constant rotation
- Wash the beads once with PBS to remove the unbound protein, and resuspend them in 50 µl of PBS buffer
- The HaloTag-SpyTag bead solution can be kept under constant rotation in a fridge until use

**Critical step:** The SpyTag-HaloTag hybridization to M-270 Amine superparamagnetic beads is not stable for extended periods of time. To ensure the maximum binding efficiency for the SpyTag-HaloTag coated superparamagnetic beads, it is recommended to keep them under constant rotation in the fridge in passivation buffer for up to 2 weeks.

### Setting up a magnetic tweezers experiment

*Protein construct incubation (timing: 35 min)*

- If using the biotin/streptavidin anchoring, prepare a ~1 nM dilution of the desired protein in PBS. If working with the SpyTag/SpyCatcher anchoring, prepare a ~100 nM dilution of the desired protein in PBS

**Troubleshooting**

**Critical step** To improve the storage lifetime of a polyprotein, it is advisable to aliquot the purified protein in single-use small volumes and store it at -80°C.

- Transfer a fluid chamber to an incubation chamber (simply a smaller humidity chamber) and tilt it
- Add ~100 µl of the protein dilution to the top well and discard the buffer at the bottom
- Add again ~100 µl of the protein dilution and recirculate the solution
- Bring the incubation chamber back to a flat position and top up the wells with the protein solution if needed. Incubate for ~30 min at room temperature
- If using M-270 streptavidin beads, prepare a 0.5 mg/mL dilution of M-270 beads on the passivation buffer, and keep it under rotation for ~30 min

**Critical step:** Do not use a vortex mixer to homogenize the superparamagnetic bead solution. Shake manually or resuspend with a pipette.

**Critical step:** BSA-passivated M-270 superparamagnetic beads can be stored for over a week under constant rotation.

- After the protein is incubated, wash the chamber with the working buffer twice.

**Critical step:** The recommended working buffer for long-term experiments is PBS, namely 50 mM sodium phosphate (Na_2_HPO_4_ and NaH_2_PO_4_), 150 mM NaCl, and 10 mM ascorbic acid pH 7.3. Other saline buffer solutions (*i.e.* Hepes, Tris) can be alternatively used, always supplemented with 10 mM ascorbic acid to minimize detrimental oxidative modifications.

*Gap alignment and magnet position (MP) adjustment (timing: 20 min)*

**Extended Figure 3:**
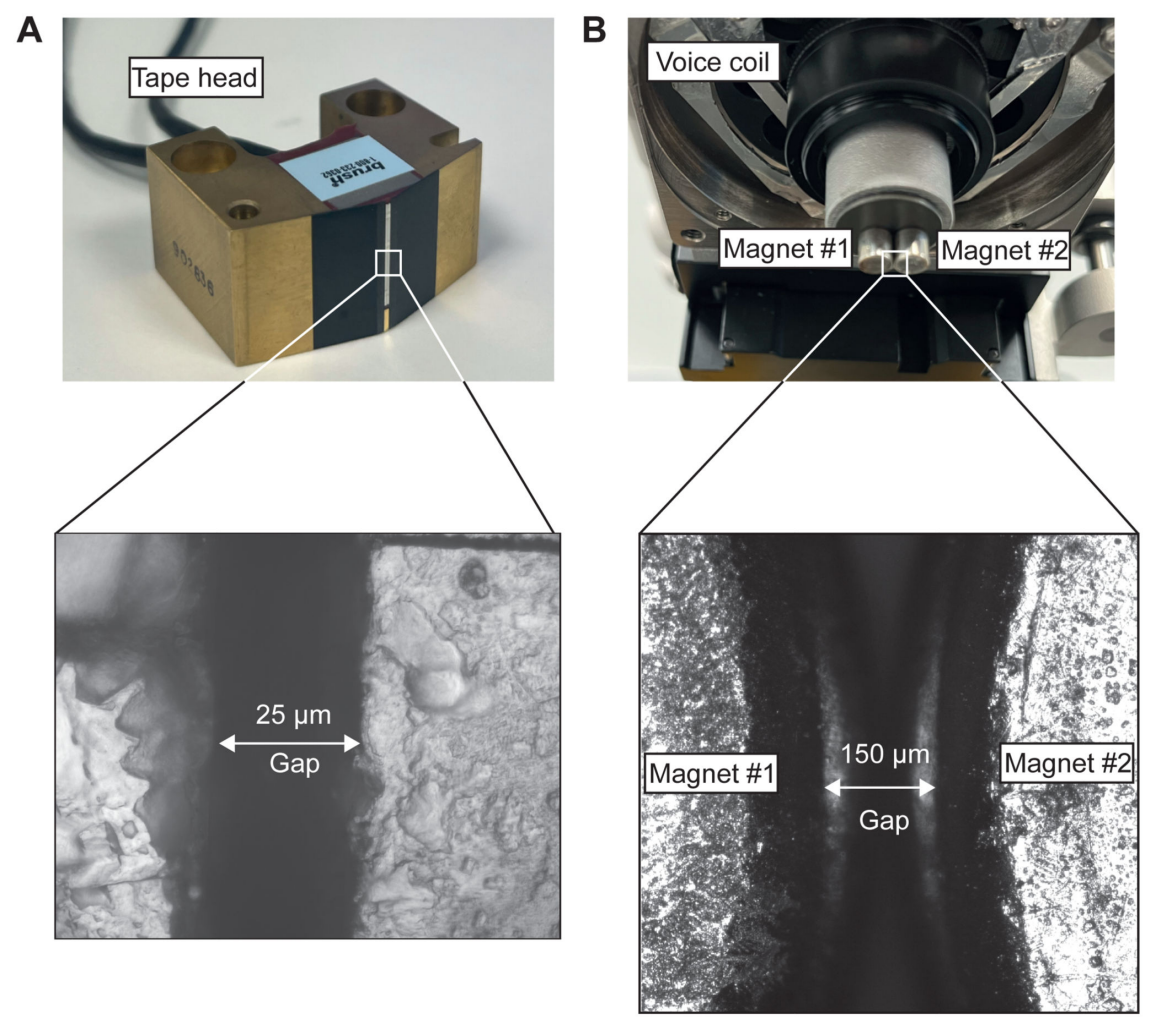
Photograph of the magnetic tape head and voice-coil-mounted permanent magnets with a magnification of the gap region.

- Before starting an experiment, it is crucial that the microscope is aligned with the gap (of either the magnets or the tape head) and that the *MP* is correctly referenced with respect to the bottom glass, in the case of the permanent magnets.
- To align the gap in the permanent magnet configuration (Extended Fig. 3), use a 10x air objective to get an image of the magnets, and move them so that the gap is placed approximately in the center of the image. In the case of the tape head, which has a much narrower gap, use the 100x oil objective to obtain an image of the tape head. The gap must be aligned in the center of the image. In case it is not, align the microscope, so the optical axis is aligned with the gap.
- To calibrate the *MP*, use a bottom cover slide of the same thickness as those employed in the fluid chamber assembly (without any chemical treatment) and place it on the microscope, drawing a line on top with a marker. Focus the microscope on the drawn line, right at its edge, and slowly bring the magnets close to the glass, this is, decreasing *MP.* When *MP*=0, the magnets should be barely touching the glass, noticeable by a slight shiver of the glass’ image. In case the magnets are too far or too close, approach or retract the voice coil to correct the offset (see Supplementary Video 3).

*Incubation of the superparamagnetic beads (timing: 5 min)*

- Once the fluid chamber is placed on the microscope under conditions of zero force (this is, with the permanent magnets moved away from the chamber or the electric current off if using the tape-head configuration), add ~30 µl of the streptavidin magnetic beads or ~10 µl of derivatized amine beads to one of the wells and recirculate the solution through the chamber to ensure that the beads flow in the fluid channel. They can be directly observed under the microscope
- Let the beads incubate without force application for ~2-5 min

**Troubleshooting**

- After the incubation time, set the force to a reference value (typically ~4 pN, but it can be a lower force in case the protein of interest has very low mechanical stability) in order to remove non-attached beads and set some tension conditions on the tethered beads to prevent physisorption of the beads to the surface

*Searching for an individual Protein of Interest (PoI) (timing 5 min to 4 hours)*

- These are some indicative criteria to identify superparamagnetic beads with a tethered protein that allow minimizing the searching time:
  - Jiggle: The superparamagnetic bead should jiggle with a small amplitude (~10% of the bead’s diameter), see Supplementary Video 1 from 0 to 9 s.
    **Troubleshooting**
  - Focal plane: The superparamagnetic bead should be on the same focal plane as the neighboring reference beads.
    **Troubleshooting**
  - Reference bead: Each superparamagnetic bead chosen for measurement should have an isolated reference bead within the field of view.
    **Troubleshooting**

- Select the magnetic and reference beads to define the region of interest (ROI), see SupplementaryVideo 1. The size of this region determines the frame rate of the experiment. Therefore, it is desirable to select a reference bead as close to the magnetic bead as possible
- Create the stack library. Since the library is built from 128 positions spaced 20 nm, the stack must start 1280 nm below the measuring position (which is when the beads are in focus). This will ensure that the stack library has enough positions below and above the measuring position
- Once the stack library has been built, bring the image back into focus (increasing the piezo position back by 1280 nm). The correlation profiles should be two clear and narrow overlapping peaks (Fig. 3D). If this is not the case, repeat the stack, starting from a different position
- To test whether the bead has a protein construct attached, a force-ramp is typically employed. The speed and force thresholds of the ramp depend on the expected mechanical stability of the protein under study
- During the force ramp, different scenarios can occur:
  - No or very little extension with no step.
    i. This typically indicates that the bead is non-specifically attached to the surface. The bead should be discarded.
  - Very long extension (>200 nm).
    ii. This is often the hallmark of some non-desired tether. The bead should be discarded.
  - Multiple steps of similar size that appear in a (more or less) reproducible way but at a force higher than expected.
    iii. This most likely corresponds to double or even triple protein tethers on the same pulling superparamagnetic bead. It could indicate that the concentration employed for incubation is too high. If several beads show this behavior, the incubation concentration should be decreased.
  - A successful protein tether is fingerprinted by a clear unfolding step (or more than a single event if the protein, for instance, unfolds through an intermediate).
    iv. This unfolding step should be reproducible, *i.e.,* after a low-force pulse to refold the protein, the unfolding event should appear again in a new force ramp.
    v. Note that some proteins might require long times to refold, so the duration of the low-force pulse should be chosen accordingly.

- Once a bead fulfills the conditions described in step 81 the experiment can start. The most frequent experimental modes are described in the anticipated results section.

*Achieving ultra-stable days-long experiments*

A main advantage of our magnetic tweezers approach is the ability to measure an individual protein for very long timescales, spanning several hours or even days. In order to achieve long-lasting measurements, the key step is to seal the fluid chamber. This is accomplished by blocking the two open wells of the fluid chamber to prevent evaporation, helping minimize long-term drift. Once a “good” protein has been identified, to achieve a long and stable (driftless) recording, the next steps should be followed:

- To seal the fluid chamber, use high-viscous silicone oil. Employing the tip of a pipette, carefully take a drop of oil and, by gravity, let it gently slip into one of the wells. Repeat on the second well. Simply by hydrophobicity, the oil will spread over the fluid on the well in a few minutes, creating a thin layer that will prevent evaporation

**Critical step:** After the chamber is sealed, no buffer exchanges can be conducted. Therefore, make sure that the desired experimental buffer is in the fluid chamber before sealing it.

- Bring the piezo scanner to its middle position (9,000 nm) and refocus manually. Re-do the stack. This will ensure that the piezo has enough range to correct any long-term drift that might appear over hours-long experiments.
- Program and apply a force protocol if different forces are to be explored over the long experiment or leave a fixed force value set if desired.

**Troubleshooting**

- Check the experiment periodically (at least once a day) to ensure that the piezo scanner remains within range. Re-stack if necessary.

**Critical step:** The limiting factors in the duration of the experiment are the possible detachment of the bead-to-molecule interaction (over very long timescales, the biotin/streptavidin interaction can break even at very low forces) or some oxidative modification that irreversibly alters the behavior of the protein, which, even when using a strong oxygen scavenger (as the recommended 10 mM ascorbic acid) can stochastically occur over very long timescales.

### Timing

Steps 1 to 8, cloning and expression of the protein constructs: 6 d

Steps 9 to 23, purification of protein constructs: 2 d

Steps 24 to 34, Cleaning of the bottom cover slides: 3 h

Steps 35 to 41, Silanization of bottom cover slides: 2 h

Steps 42 to 51, Cleaning and silanizing top cover slides: 1 h 45 min

Steps 52 to 55, Assembly of the fluid chamebrs: 15 min

Steps 56 to 63, Preparing HaloTag binding chemistry for bottom cover slides: 2 h

Step 64 to 65, HaloTag ligand binding reaction to bottom coverslide: 12 h

Steps 66 to 70, BSA-tris passivation of the fluid chambers: minimum of 3 h

Steps 71 to 75, Preparing HaloTag binding chemistry for amine based superparamagnetic beads: 1 h 30 min

Steps 76 to 77, HaloTag ligand binding reaction to amine superparamagnetic beads: 12 h

Steps 78 to 80, BSA-tris passivation of the superparamagnetic beads: 4 h 15 min

Steps 81 to 85, HaloTag/spyTag hybridization to superparamagnetic beads: 50 min

Steps 86 to 92, Protein construct incubation to fluid chambers: 35 min

Steps 93 to 95, Magnetic tweezer force check-up calibration: 20 min

Steps 96 to 98, Superparamagnetic bead binding to protein constructs: 5 min

Steps 99 to 105, looking for superparamagnetic beads/proteins of interest: from 5 min to 4 h

Steps 106 to 109, setting up days-long experiments: 10 min

### Anticipated results

When studying the nanomechanics of a single protein using magnetic tweezers force spectroscopy, there are two main experimental modes based on the force-application protocol: force-ramps and constant force experiments.

#### Force-ramp mode

In a force-ramp experiment, the force is changed linearly between two values *F*_0_ and *F*_1_, during a time ⊿*t*. Here, the control parameter is the pulling rate (in pN/s), defined as *r*_F_=(*F*_1_-*F*_0_)/⊿*t*. Force ramp experiments are useful for establishing molecular fingerprints and for characterising the mechanical stability of a protein (unfolding dynamics). When *r*_F_>0, and starting from the folded state, the protein will unfold stochastically at some force *F*, being detected as a sudden stepwise increase in the molecular extension. The size of the observed step should scale with contour length *L*_C_, considering the force at which the unfolding occurred (Eq. 3). The distribution of unfolding forces depends on the pulling rate *r*_F_, so that a higher *r*_F_ leads to higher unfolding forces, and vice-versa^77^. Assuming the Bell-Evans model^78^, the distribution of unfolding forces *p*_U_*(F)* can be described as^77^:

(6) $$p_{U}\left(F\right)=\frac{k_{U}^{0}}{r_{F}}e^{\frac{Fx_{U}^{†}}{kT}}e^{-\frac{k_{U}^{0}kT}{rF^{x_{U}^{†}}}\left(e^{\frac{Fx_{U}^{†}}{kT}-1}\right)},$$

where *kT*=4.11 pN/nm is the thermal energy, *x*^†^_U_ is the distance to the transition state for unfolding, and *k*^0^_U_ *is* the intrinsic unfolding rate.

To extract *x*^†^_U_ and *k*^0^_U_, it is best to measure the typical (most probable) unfolding force F*_U_ and plot it as a function of the pulling rate *r*_F_, which follows^77^:

(7) $$F_{U}^{*}=\frac{kT}{x_{U}^{†}}\ln\frac{r_{F}x_{U}^{†}}{k_{U}^{0}kT}$$

**Figure 7:**
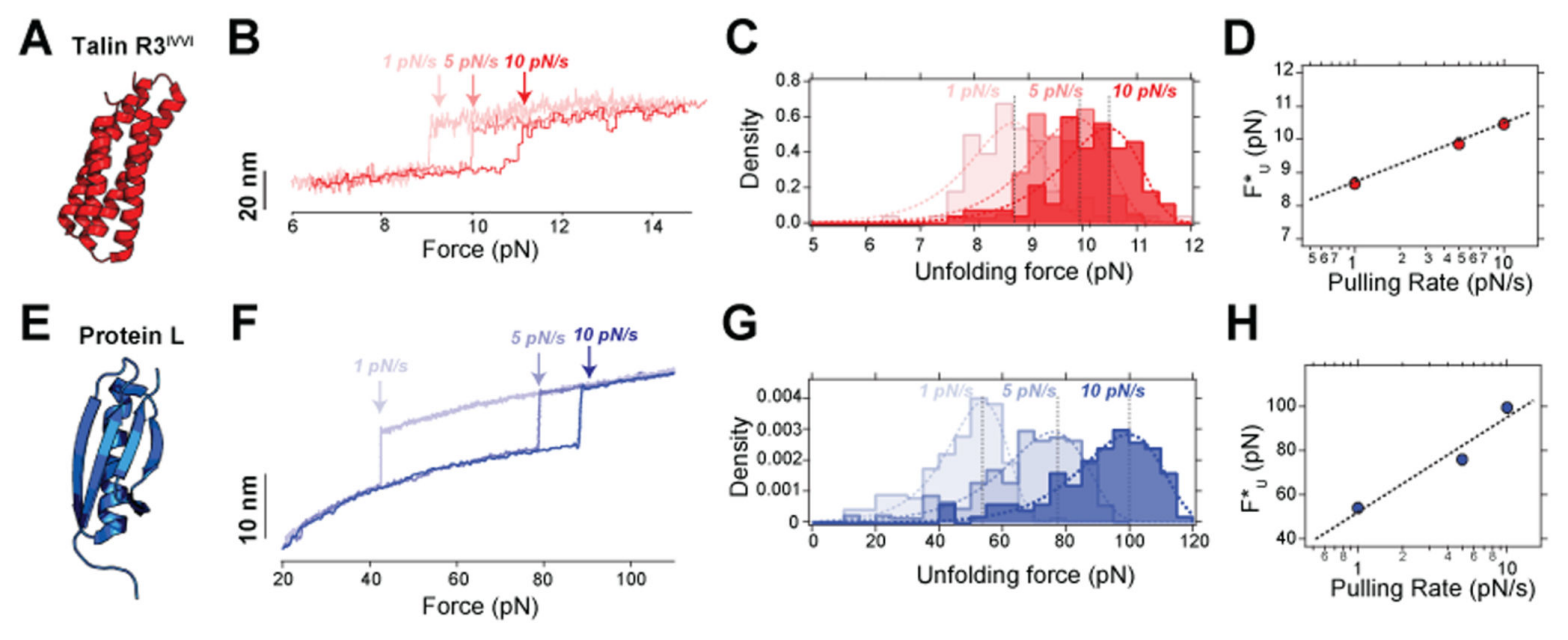
Characterization of the mechanical stability of single proteins using the force-ramp mode **(A)** Structure of the talin R3 domain (PDB: 2L7A). We use the IVVI mutant as a model protein, owing to its higher mechanical stability. Typical unfolding force-ramp trajectories of R3^IVVI^ at 1 pN/s, 5 pN/s, and 10 pN/s. The unfolding of R3^IVVI^ is characterized by a sudden ~20 nm step-wise increase in the protein’s extension. As the pulling rate is increased, the unfolding force shifts to higher values. **(B)** Distribution of unfolding forces measured at 1 pN/s, 5 pN/s, and 10 pN/s fitted to Eq. 6. Data from *N*=100 (1 pN/s), N=133 (5 pN/s), and *N*=95 (10 pN/s) unfolding events using 5 individual molecules. **(C)** Unfolding force as a function of the pulling rate. Fitting to Eq. 7, we characterise the unfolding nanomechanics of R3^IVVI^ as *k*_U_^0^=(1.62±0.10)x10^-5^ s^-1^ and *x*_U_^†^=5.33±0.21 nm. **(D)** Structure of protein L (PDB: 1HZ6) and a typical protein L unfolding trajectories at 1 pN/s, 5 pN/s, and 10 pN/s. The unfolding of protein L is chacterized by a ~15 nm step. **(E)** Distribution of unfolding forces measured at 1 pN/s, 5 pN/s, and 10 pN/s fitted to Eq. 6. Data from *N*=211 (1 pN/s), *N*=192 (5 pN/s), and *N*=100 (10 pN/s) unfolding events and 6 molecules. **(F)** Typical unfolding force as a function of the pulling rate, fitted to Eq. 7, allowing us to characterise the unfolding nanomechanics of protein L as *k*_U_^0^=(3.29±0.50)x10^-3^ s^-1^ and *x*_U_^†^=0.22±0.06 nm. All traces were smoothed with a 101-point 4^th^-order Savitzky-Golay algorithm. Error bars are SD

Here, we use two structurally and nanomechanically distinct proteins as model systems—Talin R3^IVVI^ (Fig. 7A) and Protein L (Fig. 7D)—to exemplify how to measure their mechanical stability using force-ramps. Both domains are pulled at three different pulling rates (1, 5 and 10 pN/s, Fig. 7 B,F), ensuring that the ramp’s duration is long enough to unfold the protein as, otherwise, the force distribution would be biased. A statistically robust characterization of the mechanical stability of the protein of interest requires measuring at least ~100 unfolding events per pulling rate, allowing the calculation of the unfolding force distributions (Fig. 7 C,G). Fitting the typical unfolding force (defined as the mode of the distribution, *F*_U_*) as a function of the pulling rate to Eq. 7, allows for extracting *x*^†^_U_ and *k*^0^_U_ (Fig. 7 D,H).

The distinct unfolding properties of our two model proteins are readily captured by their characterization using the force ramp mode. R3^IVVI^ shows just a small increase in its unfolding force with the pulling rate (~8 pN at 1 pN/s vs. ~11 pN at 10 pN/s, Fig. 7 C,D), which is reflected in a shallow slope in the F*_U_ vs. *r*_F_ curve, corresponding to a large distance to transition state for unfolding (*x*^†^_U_=5.3 nm), common feature of proteins with a very high force-sensitivity. By contrast, the unfolding force for protein L shifts greatly with the pulling rate (~50 pN at 1 pN/s vs. ~100 pN at 10 pN/s, Fig. 7 G,H) and shows a steep slope in the F*_U_ vs *r*_F_ dependence, so a short distance to transition state *x*^†^_U_=0.22 nm, indicative of low force sensitivity. These two scenarios are often referred to as being a compliant (large *x*^†^_U_ as R3^IVVI^) or brittle (small *x*^†^_U_ as protein L) protein, respectively. Data corresponding to force-ramp trajectories of both R3^IVVI^ and protein L is included in the Supplementary Data 1 file.

In principle, force-ramps could also be used to characterize the refolding properties of a protein, by linearly decreasing the force (*r*_F_<0) from the unfolded and stretched protein conformation, until we observe a downward step at some force *F* that fingerprints the refolding event. However, this is quite unpractical for most proteins as they show large hysteresis in their unfolding/refolding forces, which implies that the refolding event occurs at forces that are too low for the refolding step to be resolved, in particular at high pulling rates.

#### Constant force mode

In a constant force experiment, the protein is under a fixed force, which allows measuring the protein’s conformational dynamics in equilibrium. Unfolding and folding transitions are monitored as step-wise upward and downward changes in the protein’s end-to-end length, measured as a function of time. This allows characterizing the protein’s folding dynamics in terms of its folding (*r*_f_) and unfolding rates (*r*_u_), defined as *r*_f_=1/<*t*_u_> and *r*_u_=1/<*t*_f_>, being <*t*_u_> and <*t*_f_> the average residence times in the unfolded and folded states, respectively. Typically, the unfolding rates increase with force while, conversely, the refolding rates decrease with force. Noteworthy, at higher forces, *r*_u_>>*r*_f_, and once the protein unfolds, the probability of observing a refolding event over a reasonable experimental time is vanishingly small and similarly, at significantly lower forces (*r*_u_<<*r*_f_), the probability of unfolding will be negligible. For each protein, there is a certain force range whereby the unfolding and folding rates are of comparable magnitude, which allows monitoring the reversible folding dynamics of the protein under force. In this case, we can characterize the folding equilibrium as the relative population of the folded state with respect to the unfolded state (folded fraction, *P*_F_). If *P*_U_ is the probability of being in the unfolded state (*P*_U_=1-*P*_F_ in the two-state case), then, in equilibrium, we have *r*_U_*P*_F_=*r*_F_*P*_U_ (detailed balance condition), which means that we can characterize the folding equilibrium from the folding/unfolding rates as:

$$P_{F}\left(F\right)=\frac{r_{F}\left(F\right)}{r_{U}\left(F\right)+r_{F}\left(F\right)},$$

which, qualitatively, will follow a sigmoidal force-dependence —at very low forces, the unfolding rates are vanishingly small, which implies *P_F_*=1, while at higher forces the refolding rates are also vanishingly small, implying a folded fraction of *P_F_*=0.

In the simplest approximation, the force dependency of the folding *r*_F_ and unfolding *r*_U_ rates can be described by the Bell-Evans model:

(8) $$r_{F}\left(F\right)=k_{F}^{0}e^{-Fx_{F}^{†}/kT}$$

(9) $$r_{U}\left(F\right)=k_{U}^{0}e^{Fx_{U}^{†}/kT}$$

where *x^†^_F_* and *x^†^_U_* are the distances to the transition state for folding and unfolding, and *k^0^_F_ and k^0^_U_* for folding and unfolding the intrinsic rates, respectively. From this, the folded fraction:

(10) $$P_{F}\left(F\right)=\frac{1}{1+\frac{k_{U}^{0}}{k_{F}^{0}}e^{F\left(x_{U}^{†}+x_{F}^{†}\right)/kT}}=\frac{1}{1+e^{\left(F-F_{0.5}\right)/W}}$$

which is a sigmoidal function (Fermi function). Here *F*_0.5_=-*ln(k*^0^_U_*/k*^0^_F_*)kT/(x*^†^_U_*+x*^†^_F_*)* is the coexistence force, for which *P*_F_=0.5, while *W=kT/(x*^†^_U_*+x*^†^_F_*)* is the decay rate of the sigmoid.

**Figure 8:**
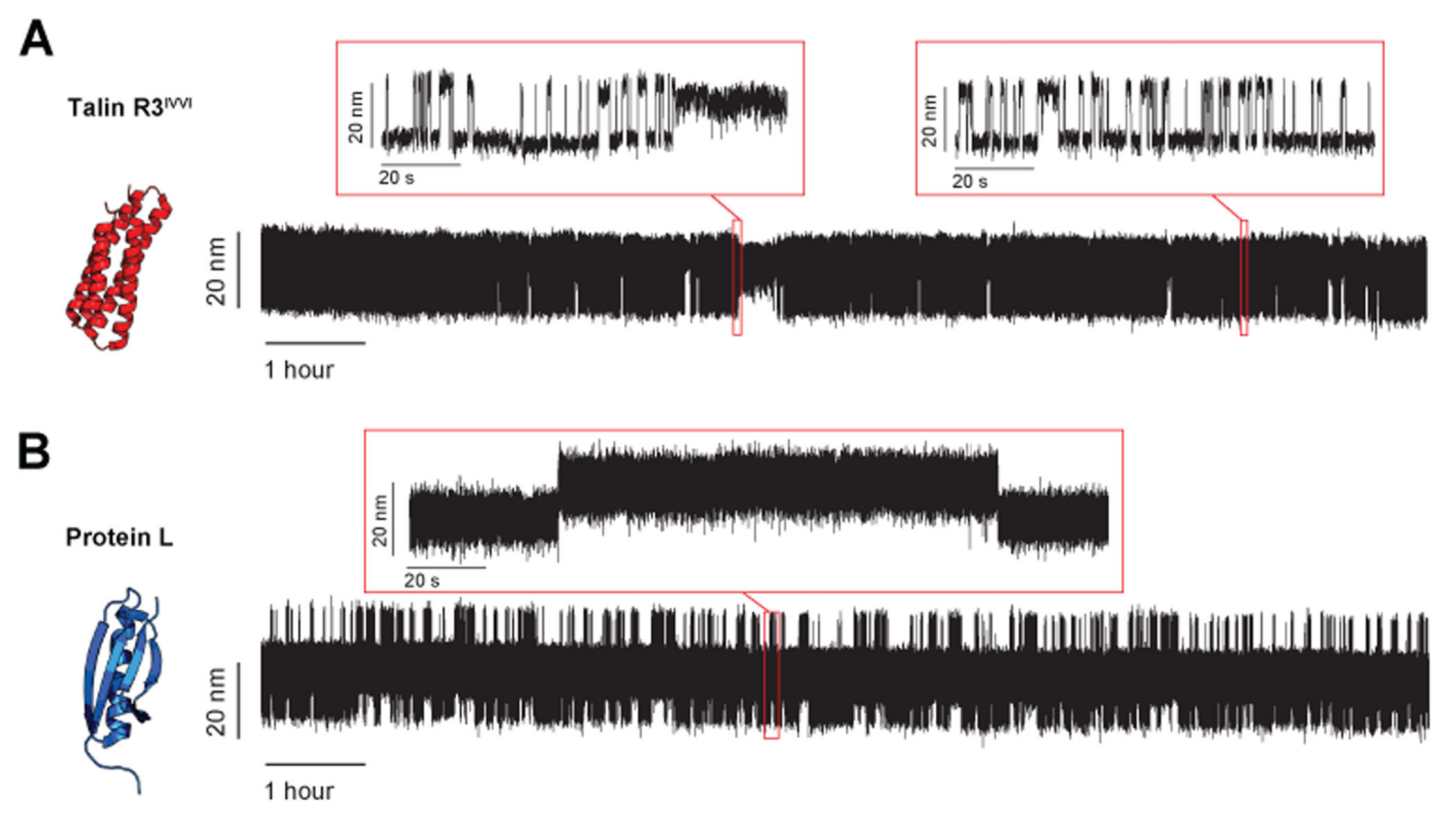
Characterization of single protein folding dynamics using the constant force mode. **(A)** Typical magnetic tweezers recordings of R3^IVVI^ pulled at 8 pN, 8.5 pN, and 9 pN. When held at a constant force, R3^IVVI^ reversibly switches between the folded state (lower extension state) and the unfolded state (higher extension state), hallmarked by a change in length of ~20 nm. As the force increases, the population quickly shifts from the folded to the unfolded state. (inset) Fragment of the 8.5 pN recording, showing the overlaid idealised trace calculated with a step-detection algorithm (thresholding). Measuring the residence times in the folded (*t*_f_) and unfolded state (*t*_u_), allows calculation of the folding and unfolding rates. **(B)** Folding (red) and unfolding (blue) rates as a function of force. The folding rates decrease exponentially with force, while the unfolding rates increase exponentially, following Bell-Evans model with *k*_U_^0^=(4.61±2.52)x10^-6^ s^-1^ and *x*_U_^†^=4.27±0.34 nm and *k*_F_^0^=(1.84±1.01)x10^6^ s^-1^ and *x*_F_^†^=6.78±0.59 nm. Data obtained from 12 independent molecules amounting *N*>10^5^ transitions. **(C)** Probability of populating the folded state (folded fraction), showing a sigmoidal shape with a coexistence force of ~8.5 pN. **(D)** Typical magnetic tweezers recordings of protein L at 7 pN, 8 pN, and 9 pN. Similar to R3^IVVI^, protein L switches reversibly between the folded and unfolded states, albeit with much slower kinetics and over a broader force range. (inset) Fragment of the 8.5 pN recording, showing the overlaid idealised trace calculated with a step-detection algorithm (thresholding). **(E)** Folding (red) and unfolding (blue) rates of protein L as a function of force, respectively decreasing (folding) and increasing (unfolding) exponentially with force. In contrast to R3^IVVI^, the folding and unfolding rates have very different slopes (different force sensitivity) that hallmark the very different nanomechanical properties of both proteins. From fits to the Bell-Evans model, we obtain *k*_U_^0^=(3.10±0.06)x10^-3^ s^-1^ and *x*_U_^†^=0.30±0.03 nm and *k*_F_^0^=(8.96±0.05)x10^3^ s^-1^ and *x*_F_^†^=7.51±0.38 nm. Data obtained from 12 individual molecules and *N*>10^3^ transitions. **(F)** Folded fraction of protein L as a function of force, showing a sigmoidal dependence with a coexistence force of ~8 pN, similar to R3^IVVI^ but broader, indicating a shallower force dependence. All traces were smoothed with a 101-point 4^th^-order Savitzky-Golay algorithm. Error bars are SD.

Again, here we use the same two model proteins—talin R3^IVVI^ and Protein L—to illustrate how to mechanically characterize the folding dynamics using constant force pulses. Usually, 7 to 10 different constant forces are employed to characterize the behavior of a protein of interest (Fig. 8). The explored range of forces and the measuring time windows at each of these forces depends on the nanomechanical properties of the protein under study. Coexisting folding/unfolding transitions are observable only when the folding and unfolding rates are comparable (typically within an order of magnitude of difference, otherwise, the slower transition becomes unpractical to observe within a reasonable measuring time). Hence, at higher forces, only the unfolding rates can be characterized, while at lower forces, just the folding rates.

After obtaining the different force pulses, a step-detection algorithm is used to idealize the single protein recordings and automatically detect the folding and unfolding transitions to measure the residence times in the folded (*t*_f_) and unfolded state(*t*_u_) (red lines, insets, Fig. 8 A,D). There is a large number of available methods for detecting transitions in noisy time series. In magnetic tweezers, provided the generally high signal-to-noise ratio, a simple thresholding algorithm is sufficient. If a protein shows a low signal- to-noise ratio (i.e., the extension changes are small compared to the molecular noise), more sophisticated methods such as Hidden Markov modeling^25,79,80^ can be employed.

From the dwell time distributions, the folding and unfolding rates at each force can be calculated (Fig. 8 B,E), as well as the folded fraction (Fig. 8 C,F). Finally, Eqs. 8 and 9 can be used to fit the rates versus force dependence and obtain *x*^†^_U/F_ and *k*^0^_U/F_, while Eq. 10 can be used to extract *F*_0.5_. Of note, the Bells-Evans model, provides the simplest description for a protein’s (un)folding kinetics as a function of the force. When exploring the (un)folding dynamics of a protein over a very broad range of forces deviations from this simple exponential approximation might arise. This is typically because, assuming a one-dimensional description of the protein’s free energy landscape, the position of the free energy barrier shifts with force in a measurable way. More accurate models have been developed to account for the different shapes of the energy barrier, and their deformability with force (see, for instance, refs^23,81,82^).

The constant force experiments (Fig. 8) also reflect the different nanomechanical properties of talin R3^IVVI^ and protein L. R3^IVVI^ shows reversible folding dynamics over a narrow force range (~7.5-9.5 pN) and a symmetric force dependence of its folding and unfolding rates (Fig. 8B), which gives rise to very similar distances to transition state for folding and unfolding (*x^†^_F_*=6.8 nm; *x^†^_U_*=4.3 nm, respectively). By contrast, protein L exhibits much slower folding dynamics, implying a broader force range over which folding can be observed (~4-10 pN). Consequently, the refolding and unfolding rates have very different force dependencies (Fig. 8E), with a much larger distance to the transition state for refolding than for unfolding (*x^†^_F_*=7.5 nm; *x^†^_U_*=0.3 nm).

**Figure 9:**
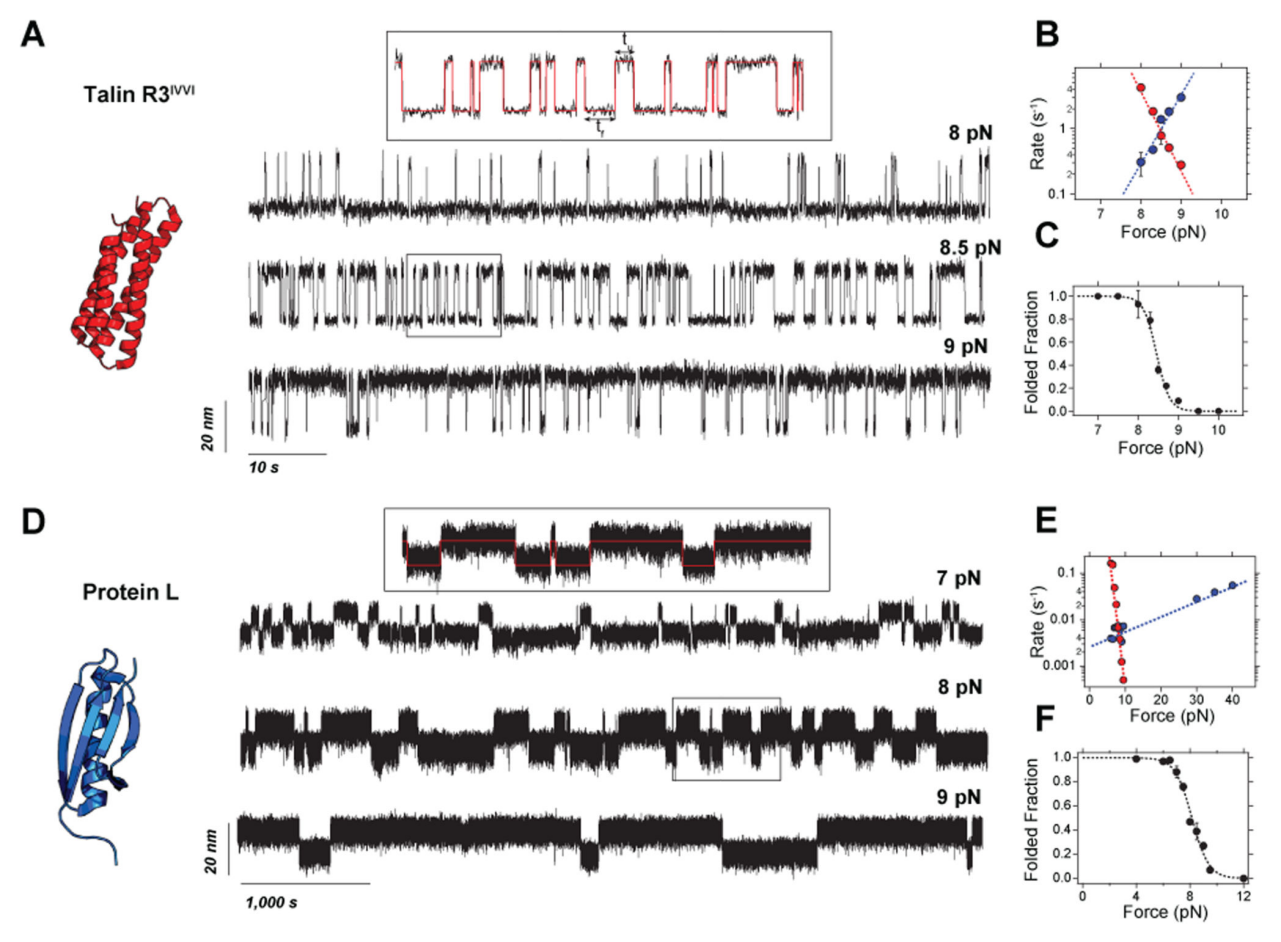
Measuring long-equilibrium dynamics of individual proteins under force. 12-hour magnetic tweezers recording of talin’s R3^IVVI^ domain at 8.5 pN (**A**) and protein L at 8 pN (**B**). The insets show a detail of the protein’s dynamics. In the case of R3^IVVI^, the long-lasting recordings unveil the appearance of rare conformational states, while protein L maintains its two-state folding dynamics. Data smoothed with a 101-point 4^th^-order Savitzky-Golay algorithm. No drift correction has been applied.

Figure 9 shows typical long recordings of R3^IVVI^ and protein L (12 hours long in this case) at constant force. Achieving such long recordings can be useful for observing low-probability molecular events occurring over long timescales, such as in the case of the rare conformational states visited by talin’s R3^IVVI^ (Fig. 9A).

#### Fluctuation analysis method to fingerprint protein conformational states

While average sudden end-to-end changes are the most frequently measured quantity in single-molecule experiments, the ability to acquire long equilibrium recordings at physiologically-low forces allows us to also monitor changes in the molecular noise. These fluctuations (or variance) in the end-to-end protein extension contain additional information on the structural dynamics of the underpinning conformational state, particularly relevant to fingerprint rare conformations different than the canonical folded/unfolded states (Fig. 9A). Additionally, this approach can be used as a method to detect protein binding events.

**Figure 10:**
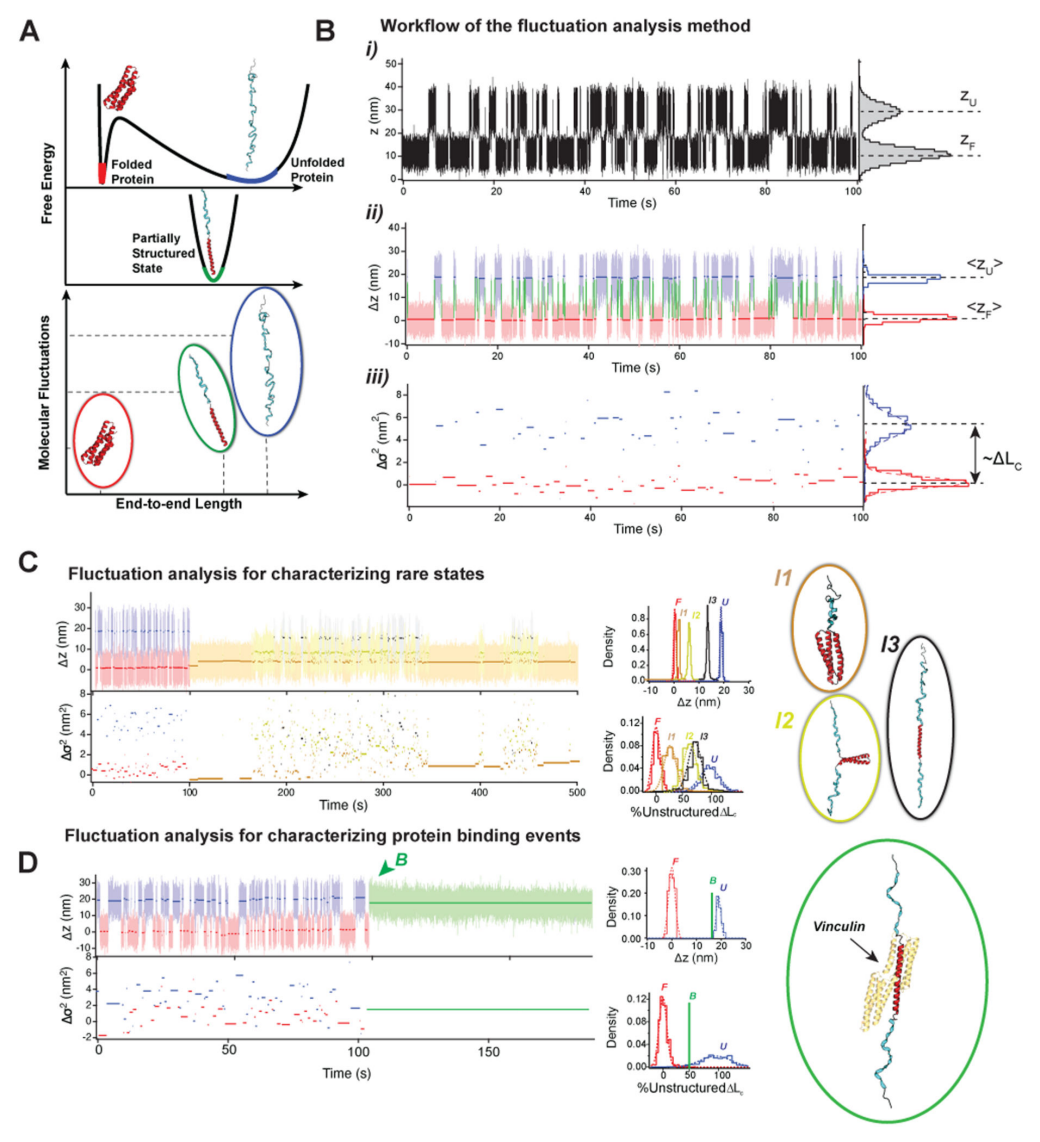
Fluctuation analysis method to fingerprint protein conformations. **(A)** Schematic conceptualization of the fluctuation analysis method. Upon unfolding, a protein becomes an unstructured polypeptide, resulting both in an increase of its end-to-end length and its molecular fluctuations. In a free-energy diagram (upper panel), this is reflected in a transition from a narrow minimum characterizing the folded state (red) to a wider basin representing the unfolded state (blue). If the protein acquires some conformational state composed of a combination of structured (red) and unstructured segments (green), its end-to-end is likely to be large, and only slightly shorter than that of the unfolded state, while its fluctuations would be greatly reduced. Therefore, the end-to-end length and molecular fluctuations can be employed in combination to fingerprint protein conformational states (lower panel). **(B**) Workflow of the fluctuation analysis method. **(i)** Raw fragment of a magnetic tweezers recording for R3^IVVI^ pulled at 8.5 pN and extension histogram indicating the end-to-end length of the folded (*z*_F_) and unfolded (*z*_U_) states. **(ii)** Analyzed recording highlighting the fragments of the trajectory assigned to the folded (red) and unfolded (blue) states, alongside the transition paths (green) between them. Histograms (right) indicate the average extension of the folded (<*z*_F_>) and unfolded (<*z*_U_>) conformations based on the average extension of each fragment. **(iii)** Difference in variance for each fragment in the folded (red) and unfolded (blue) states. The difference between the average fluctuations between states is proportional to the change in exposed contour length in each state, following Eq. 11. **(C)** Fluctuation analysis applied to a rare (low probability) conformational state in R3^IVVI^, consisting of three different conformations characterized by end-to-end extensions ranging between those of the folded (**F**) and unfolded (**U**) states. Analysis of the molecular fluctuations allows estimation of the fraction of unstructured contour length of each conformation, enabling to propose plausible and compatible protein structures. (**D**) Fluctuation analysis as a method for detecting cryptic binding events. Upon binding of an interacting protein to the mechanically unfolded and stretched state (here vinculin), the substrate protein (R3^IVVI^) undergoes a conformational change triggered by the binding event (indicated by an arrow). This new bound conformation (B) is detectable by a small change in the protein’s end-to-end length (~3 nm) and by a large change in its molecular fluctuations, which are reduced by ~50%. This is suggestive of a conformation where half of the protein’s contour length is trapped by the bound molecule (right).

From a conformational perspective, any (partial) unfolding event that releases a protein fragment from its folded conformation results in an increase of the protein’s contour length. For example, if the protein unfolds completely, the new unfolded state *U* is fingerprinted by the protein’s full contour length—which will be fully unstructured; however, some (intermediate or partially unfolded) states might be composed of a combination of unstructured and structured fragments. This will give rise to a partial change in the average end-to-end length of the protein but also to a measurable change in the end-to-end fluctuations (Fig. 10A). This is because the end-to-end length of a stretched polymer fluctuates stochastically and, similar to the average extension (Eq. 3), the variance of the extension can also be modeled with the FJC^52^. In particular, for some conformation *X*, if ⊿*σ*^2^_X_ is the change in variance with respect to the folded state, the FJC model predicts:

(11) $$\Delta \sigma_{X}^{2}=\Delta L_{C}\left[1-\coth^{2}\left(\frac{Fl_{K}}{kT}\right)+{\left(\frac{kT}{Fl_{K}}\right)}^{2}\right],$$

where *l*_K_ is the Kuhn length, and ⊿*L*_C_ is the change in contour length of the protein, released upon the conformational change that brings the protein from the folded state *F* to conformation *X*. For the unfolded state, ⊿*L*_C_ is the total change in contour length change of the protein upon mechanical unfolding and stretching (protein contour length minus the size of the folded state).

However, if the protein visits a partially unfolded state (composed of both structured and unstructured fragments), its average end-to-end length could not necessarily correlate with the released contour length (a structured fragment has a smaller end-to-end length than the equivalent unstructured polymer), but the end-to-end fluctuations will (assuming that structured segments do not or minimally contribute to the fluctuations). Therefore, by analyzing the molecular fluctuations of a protein’s particular conformation, we can infer its underpinning structural properties, and namely the fraction of the protein’s contour length that is unstructured. Noteworthy, the method can be extended to measuring protein-protein interactions since protein binding might result in a change in the protein’s intrinsic fluctuations at a given force.

Figure 10B shows the workflow for the protein fluctuation analysis methodology exemplified for the folded-unfolded dynamics of the model talin’s R3^IVVI^, while Fig. 10 C shows the method applied to a rare conformation different from the unfolded state. Finally, Fig. 10D exemplifies how the method can be applied to the detection of binding events. Raw data for the two-state folding dynamics of R3^IVVI^ and a detailed step-by-step description of the fluctuation analysis with analysis scripts for Igor Pro are included in the Supplementary Data 2 file.

1) Using the raw recording *z(t)*, identify the average extension of each conformation, here the folded <*z*_F_> and unfolded state <*z*_U_> from histograms to the protein’s extension time series (***i***). It is important to use the raw recording, as in the non-smoothed, for the the fluctuation analysis.
2) Subtract <*z*_F_> from *z*(*t*) to have the average extension of the folded state as a reference. Based on the average end-to-end length of each state, use a state-detection algorithm to identify the segments of trajectory corresponding to each state and the transition paths between them. In Fig. 10B, this corresponds to folded (red), unfolded (blue), and transition path (green). Calculate the average extension of each segment
3) Calculate the variance of each segment and subtract that of the folded state *σ*^2^_F_ from *σ*^2^(*t*) to obtain the fluctuations of the folded state as a reference. Calculate the change in variance with respect to the folded state ⊿*σ*^2^ from histograms of the fluctuations in each state. Δ*σ*^2^ is proportional to the change in structured contour length following Eq. 11. In the case of the unfolded state, ⊿*σ*^2^_U_=5.1 nm^2^, which corresponds to Δ*L*_C_=42 nm, in agreement with the contour length change expected for talin R3^IVVI^
4) For rare conformations such as the one illustrated in Fig. 10C, the procedure is alike, and a trajectory fragment exhibiting two-state unfolding dynamics from the same recording must be employed to obtain the reference conformational properties of the folded state (<*z*_F_> and *σ*^2^_F_). In the showcased state, there are three different conformations (*I1*, brown; *I2*, yellow; *I3*, black), each characterized by different changes in their average end-to-end lengths and molecular fluctuations, compatible with the proposed protein structures depicted in the right panel
5) The fluctuation analysis method can also be implemented to detect cryptic binding events, provided binding to a cryptic site will trigger a conformational change in the substrate protein polymer. We here illustrate this with vinculin binding to the talin R3^IVVI^ domain (Fig. 10D). Upon vinculin binding, talin stops folding, such that talin gets locked in a new conformation (*B*) characterized by a ~3 nm reduction in the end-to-end length from the unfolded state and a decrease by ~50% of its molecular fluctuations. This implies that half of talin’s contour length is in a structured state, trapped by the bound vinculin. This agrees with a conformation where the two vinculin binding sites are in a helical state, with vinculin bound to them, while the rest of the protein is unstructured, as illustrated in the right panel^53,54^.

### Extended Data

### Supplementary Material

Supplementary Video 1

Supplementary Video 2

Supplementary Video 3

## Figures and Tables

**Table 1:** Troubleshooting table

Step	Problem	Possible reason	Possible solution
57	The glutaraldehyde solution does not flow through the chamber	The top glass is not hydrophobic	Prepare new top glasses (steps 42-51), extending the incubation time with the repel silane (step 47)
60	There are too many clusters of references beads in the fluid chambers.	Reference beads tend to aggregate when stored for a long time.	To achieve a disperse distribution of polystyrene reference beads inside the chamber, vortex the bead solution bottle before preparing the aliquot, and sonicate the aliquot solution for a few seconds before adding it to the fluid chambers
70	Passivated fluid chambers stop working after stored for some time	The fluid chambers have dried out while being stored, or the O4 ligand has lost reactivity	The functionalized and passivated fluid chambers can be stored in the fridge for up to a month if kept at all time hydrated. Check them regularly and top up the wells with the passivation buffer if needed.
86	Superparamagnetic beads don’t get attached after protein construct incubation	The concentration of the incubated protein construct is too low	The ~1 nM/~100 nM protein dilution is an orientative concentration that works well for most Halotagged proteins. However, the optimal working concentration could depend on specific protein preparation.
97	Upon addition of the superparamagnetic beads to the fluid chamber, they get readily attached to the surface, showing no lateral fluctuations	(i) The concentration of the incubated protein is too high (too many proteins attach to the same superparamagnetic bead). (ii) The protein’s storage time has exceeded. (iii) The passivation process has not been successful	Reduce the protein concentration. If the problem persists, use a new batch of fluid chambers, blocked with fresh passivation buffer. If the problem still persists, make a new protein preparation (expression and purification).
99 a	The superparamagnetic bead does not show lateral fluctuations (no jiggling)	The bead is stuck on the surface through some non-specific interactions (i.e., physisorption).	Look for another magnetic bead showing lateral fluctuations.
99 b	The superparamagnetic bead is at a different focal plane than the neighbor reference beads.	The superparamagnetic bead has a long tether, which is not single protein	Look for another superparamagnetic bead.
99 c	The density of reference beads is very low, and it is not possible to find reference beads close to the superparamagnetic bead	(i) The glutaraldehyde stock solution has expired (ii) The stock solution of reference beads was not vortexed/sonicated properly (see troubleshooting 60) c) Steps 61 to 64 were not successful.	If the problem persists amongst the same batch of fluid chambers, prepare new chambers taking into account (i), (ii) and (iii).
109	The data visualization software crashes or cannot load the recording.	The binary data file it is too large to be processed.	Reset the saved file every ~24 h, as it can become too heavy to handle (a 24-hour-long experiment can typically have >86x10^6^ time marks).

## Data Availability

Example data from Fig. 7 and 10 can be found as Supplementary Data. Modified pFN18a plasmids from Fig. 5 are available in Addgene (pFN18A-HaloTag-Biotin: Addgene plasmid #206039; pFN18A-HaloTag-SpyCatcher Addgene plasmid #206041). Other data that supports the plots within this paper are available from the corresponding author upon reasonable request.

## Editorial Summary

-We use short summaries (up to 250 characters) to highlight all our protocols on our home page. This will be used to summarize your protocol on our homepage. Please check this summary is accurate.

Ultra-stable magnetic tweezers allow measuring individual protein dynamics in equilibrium under physiologically relevant pulling forces and over long-timescales of days to weeks, enabling high-precision molecular studies in mechanobiology.

## Key Points

2 Key points, with a total length of 70 words (written by the editor and, in the online version of the article, presented after the Abstract and before the Introduction).

- Ultra-stable magnetic tweezers for measuring the conformational dynamics of individual proteins at physiologically relevant low forces and over long timescales.

- Magnetic fields are created using either permanent magnets or a tape head, which generates precisely calibrated forces for pulling single proteins tethered between a superparamagnetic bead and a functionalized glass substrate.

## Code availability

Scripts for the fluctuations analysis are included in the Supplementary Data. Data acquisition code can be accessed at https://doi.org/10.5281/zenodo.8092186.
